# Assessment of a Standardized Pre-Operative Telephone Checklist Designed to Avoid Late Cancellation of Ambulatory Surgery: The AMBUPROG Multicenter Randomized Controlled Trial

**DOI:** 10.1371/journal.pone.0147194

**Published:** 2016-02-01

**Authors:** Sonia Gaucher, Isabelle Boutron, Florence Marchand-Maillet, Gabriel Baron, Richard Douard, Jean-Pierre Béthoux, AMBUPROG Group Investigators

**Affiliations:** 1 Université Paris Descartes, Paris Sorbonne Cité, Paris, France; 2 Service de Chirurgie Générale, Plastique et Ambulatoire, Assistance Publique-Hôpitaux de Paris, Hôpitaux Universitaires Paris Centre, Hôpital Cochin, Paris, France; 3 Centre d'Épidémiologie Clinique, Hôpital Hôtel Dieu, Assistance Publique des Hôpitaux de Paris, Paris, France; 4 INSERM, UMR 1153 Epidemiology and Statistics Sorbonne Paris Cité Center (CRESS), METHODS team, Paris, France; 5 Unité de Chirurgie Ambulatoire, Pôle Digestif-Anesthésie, Assistance Publique-Hôpitaux de Paris, Hôpitaux Universitaires Paris Est, Hôpital Saint-Antoine, Paris, France; 6 Service de Chirurgie Générale et Digestive, Hôpital Européen Georges Pompidou, Paris, France; University Hospital Oldenburg, GERMANY

## Abstract

**Objectives:**

To assess the impact of a standardized pre-operative telephone checklist on the rate of late cancellations of ambulatory surgery (AMBUPROG trial).

**Design:**

Multicenter, two-arm, parallel-group, open-label randomized controlled trial.

**Setting:**

11 university hospital ambulatory surgery units in Paris, France.

**Participants:**

Patients scheduled for ambulatory surgery and able to be reached by telephone.

**Intervention:**

A 7-item checklist designed to prevent late cancellation, available in five languages and two versions (for children and adults), was administered between 7 and 3 days before the planned date of surgery, by an automated phone system or a research assistant. The control group received standard management alone.

**Main Outcome Measures:**

Rate of cancellation on the day of surgery or the day before.

**Results:**

The study population comprised 3900 patients enrolled between November 2012 and September 2013: 1950 patients were randomized to the checklist arm and 1950 patients to the control arm. The checklist was administered to 68.8% of patients in the intervention arm, 1002 by the automated phone system and 340 by a research assistant. The rate of late cancellation did not differ significantly between the checklist and control arms (109 (5.6%) vs. 113 (5.8%), adjusted odds ratio [95% confidence interval] = 0.91 [0.65–1.29], (p = 0.57)). Checklist administration revealed that 355 patients (28.0%) had not undergone tests ordered by the surgeon or anesthetist, and that 254 patients (20.0%) still had questions concerning the fasting state.

**Conclusions:**

A standardized pre-operative telephone checklist did not avoid late cancellations of ambulatory surgery but enabled us to identify several frequent causes.

**Trial Registration:**

ClinicalTrials.gov NCT01732159

## Introduction

Cancellation of operations on or shortly before the scheduled date results in wasted resources, reduced operating room efficiency and delayed patient care, and can also have a negative impact on staff motivation, patient satisfaction and hospital profitability. Large cohort studies have shown late cancellation rates of between 5% and 18% for inpatient and/or ambulatory surgery [1–5].

Cancellations may be due to patient-related factors (e.g. unilateral decisions to postpone or suspend surgery, non-attendance, forgetfulness, intercurrent illness, or poor adherence to medical instructions), unavailability of preoperative tests, or logistic issues (scheduling errors, lack of specific equipment, information breakdown, etc.). Patient-related factors are reported to be the most common reasons for cancellation [1–7].

Late cancellations are particularly problematic in ambulatory surgery units (ASUs) [8]. Indeed, their organization is based on the stepwise “clinical pathway” concept, from the first consultation with the surgeon to a home call the day after surgery. Thus, reducing the rate of late cancellations is a major challenge for ASUs [9].

We postulated that a standardized pre-operative telephone checklist administered to each patient by an automated phone system a few days before surgery could help to reduce the number of late cancellations in multidisciplinary ASUs located in Paris hospitals. Such a checklist would need to be sufficiently broad to cover all surgical settings (adult and pediatric patients, small and large ASUs, all surgical specialties). It should also be computerized, inexpensive, and require little ASU staff intervention.

## Methods

### Study design

We developed a pre-operative telephone checklist and tested it in 11 multidisciplinary ASUs, in a two-arm, parallel-group, multicenter randomized controlled trial (AMBUPROG). The ethics committee Hôtel-Dieu France 1 that approved the AMBUPROG protocol, agreed that verbal consent was sufficient. The study was planned, conducted and reported according to the Consolidated Standards of Reporting Trial Guidelines for trials of non-pharmacologic treatments [10–11]. The protocol for this trial and supporting CONSORT checklist are available as supporting information; see S1 CONSORT Checklist and S1–S8 Protocols.

### Randomization

#### Sequence generation

Randomization was computer-generated and managed by an independent statistician from the Cochin Clinical Epidemiology center using Cleanweb statistical software. The unit of randomization was the patient, and the allocation ratio was 1:1. Randomization was stratified by center in blocks of variable size.

#### Allocation concealment

The sequence was concealed by a computer interface. Each patient's allocation was revealed on logging in to the study’s secure website.

#### Implementation

Only the independent statistician and computer programmer had access to the randomization list. The statistician provided the list to the programmer, who uploaded it to the study’s secure website. The list was not available to the persons enrolling patients.

### Blinding

Blinding was not possible in this study, but the data collectors were not aware of the arm to which each patient had been allocated.

### Setting and participants

#### The ASUs

The study was conducted in eleven multidisciplinary ASUs located in eleven Parisian university hospitals. Nine ASUs managed both adults and teenagers (Port-Royal, Saint-Antoine, Jean Verdier, Tenon, Georges Pompidou, Ambroise Paré, Bicêtre, Bichat, and Avicenne), whereas two were exclusively pediatric (Trousseau and Robert Debré).

Eight of the ASUs were autonomous, with their own patient pathway, while three were embedded within conventional surgical departments. Five ASUs operated on fewer than 50 patients per week (Port-Royal, Jean Verdier, Tenon, Ambroise Paré, Trousseau).

To avoid overrepresentation of patients from the largest ASUs (Saint Antoine and Georges Pompidou), recruitment by these two ASUs was randomly restricted to every second week from May 2013 to September 2013.

#### Patients

Patients were eligible if they were scheduled for ambulatory surgery under general anesthesia, locoregional anesthesia and/or sedation within the next 30 to 7 days and could be reached by telephone. Patients were not eligible if they were undergoing emergency ambulatory surgery, local anesthesia (whatever the indication), non-surgical gastrointestinal endoscopy, or termination of pregnancy. Verbal consent was obtained from the informed patients or from their parents/guardians in case of children. They could withdraw the study, at any moment, by calling a toll free number available 24/24 hours 7/7.

### Intervention

#### Development of the intervention

The aim was to develop a simple, inexpensive intervention to avoid late cancellations of ambulatory surgery in ASUs with broad patient recruitment and a wide variety of surgical specialties.

Given the reported effectiveness of checklists in reducing surgical complications and mortality [12], we developed a standardized pre-operative checklist that could be delivered to each patient via an automated phone system.

The checklist was developed in 4 phases. First, each ASU made a list of their main causes of patient-related late cancellation. Then, a series of meetings was organized to discuss these causes and to agree on the items to be included on a preliminary checklist, along with corrective actions. Each item was to concern a specific problem and solution, without generating additional work for the ASU staff. Regarding the need for an overnight fast, for example, the patient would be asked if he or she had any questions about this instruction and, if so, would be asked to contact the ASU as quickly as possible. Two versions were developed, one for adults and one for pediatric patients.

The checklist was pilot-tested on twelve caregivers who had not directly been involved in its drafting, and on twelve volunteer patients. It was then modified according to their comments and suggestions.

#### Contents of the checklist

The checklist consisted of 7 items and their corrective actions (Table 1), and was made available in five languages (French, English, Portuguese, Chinese and Arabic) and two versions (pediatric and adult).

**Table 1:** The AMBUPROG checklist.

Items	Checklist^a^	Corrective action
**1**	A^b^: You are scheduled for ambulatory surgery in the next few days. Do you confirm the date of your surgery?	Press 1 for yes, press 2 for no, press 3 to listen to the question again. If you answered yes: press 1 to confirm, press 2 to correct. If you answered no: press 1 to confirm, press 2 to correct. Please contact your surgical unit to cancel surgery.
	C^c^: Your child is scheduled for ambulatory surgery in the next few days. Do you confirm the date of your surgery?	Press 1 for yes, press 2 for no, press 3 to listen to the question again. If you answered yes: press 1 to confirm, press 2 to correct. If you answered no: press 1 to confirm, press 2 to correct. Please contact your surgical unit to cancel surgery.
**2**	A: Do you have an accompanying adult to take you home?	The operation cannot take place if you do not have an accompanying adult, and the surgery is therefore likely to be cancelled. If you cannot find one, please inform your surgical unit as soon as possible (before the eve of surgery).
	C: Do you have an organized return journey with two accompanying adults (both parents or one parent and an accompanying adult) and the presence of one adult at least on the first night after the surgery?	The operation cannot take place if you do not have two accompanying adults, and the surgery is therefore likely to be cancelled. If you cannot find two, please inform your surgical unit as soon as possible (before the eve of surgery).
**3**	A: Have you completed the administrative procedure for your admission?	If the admission formalities are not completed before the operation, the surgery will have to be postponed or even cancelled. To complete these formalities you need to bring several documents, including an ID card; a social security card, a certificate of social security coverage, a complementary health care certificate or a free universal health care certificate; parental consent for surgery on a minor or on a person of any age who is under protection. If you cannot complete your admission procedure for reasons of cost, please contact your surgical unit as soon as possible.
	C: Have you completed the administrative procedure for admission of your child?	If the admission formalities are not completed before the operation, the surgery will have to be postponed or even cancelled. To complete these formalities you need to bring several documents, including an ID card; a social security card, a certificate of social security coverage, a complementary health care certificate or a free universal health care certificate; parental consent for surgery on a minor or on a person of any age who is under protection. If you cannot complete the admission procedure of your child for reasons of cost, please contact your surgical unit as soon as possible.
**4**	A: Has your health changed since your last hospital consultation, with a possible change in your regular treatment?	Please call your surgical unit as soon as possible to check that the disease and new treatment will not interfere with the operation.
	C: Has the health of your child changed since his or her last hospital consultation, with a possible change in the regular treatment?	Please call your surgical unit as soon as possible to check that the disease and new treatment will not interfere with the operation.
**5**	A: Has the doctor or surgeon asked you to change your regular treatment in preparation for surgery?	
	5b: If so, do you have any questions about the changes in your treatment?	Please call your surgical unit as soon as possible.
	C: Has the doctor or surgeon asked you to change the regular treatment of your child in preparation for surgery?	
	5b: If so, do you have any questions about the changes in your child’s treatment?	Please call your surgical unit as soon as possible.
**6**	A: Has the doctor or surgeon asked you to undergo tests in preparation for surgery?	
	6b: If so, have you had the tests?	You must bring the results of these tests with you on the day of surgery. These tests have been prescribed because they are necessary for the operation; if they are not done, the operation is likely to be cancelled. If you are unable to have the tests, please contact your surgical unit as soon as possible.
	C: Has the doctor or surgeon asked for your child to have tests in preparation for surgery?	
	6b: If so, have the tests been done?	You must bring the results of these tests with you on the day of your child's surgery. These tests have been prescribed because they are necessary for the operation; if they are not done, the operation is likely to be cancelled. If your child is unable to have the tests, please contact your surgical unit as soon as possible.
**7**	A: Do you have any questions about the instruction to stay on an empty stomach?	Please contact your surgical unit as soon as possible for information on this instruction.
	C: Do you have questions about the instructions for your child to stay on an empty stomach?	Please contact your surgical unit as soon as possible for information on this instruction.

^a^ The checklist was provided in five languages (French, English, Portuguese, Chinese, and Arabic) and two versions (one for adults and one for children).

^b^ A: Adult.

^c^ C: Children.

#### Administration of the checklist

The checklist was administered between day 7 and day 3 before the scheduled date of surgery, by an automated phone system, between 8:30 and 20:30 from Monday to Friday, and between 10:00 and 16:00 on Saturday and Sunday. For patients who did not want to respond to the automated system, and for those the automated system failed to contact after 3 attempts, the checklist was administered by a research assistant (three attempts per day between 9:00 and noon and between 14:00 and 17:00).

### Comparator

Patients allocated to the control group received standard management alone.

### Other interventions

All the ASUs, except the Avicenne ASU, already routinely phoned patients to confirm the surgery the day before.

### Outcomes

The primary endpoint was the percentage of late cancellation, defined as cancellation the day before surgery or on the day of surgery.

Secondary endpoints were the following:

- The percentage of cancellations the day before surgery;
- The percentage of cancellations on the day of surgery;
- The percentage of conversion to conventional hospitalization.

### Sample size

A standard sample-size approach, based on a two-sided alpha risk of 5% and a statistical power of 95%, would have required 1193 subjects per group to detect a minimal clinically important difference of 6% in the cancellation rate between the intervention and control groups (4% in the intervention group and 10% in the control group). Taking into account erroneous inclusions and clustering due to a center effect, the estimated number of patients to be enrolled was increased by 70%, to 2045 patients per group.

### Statistical analysis

The study population consisted of all randomized patients minus erroneous inclusions. Mixed-effects logistic regression analysis was used to model binary primary and secondary outcome variables. The model included the intervention as the fixed effect of interest and a random center effect and intervention by center interaction entered to adjust standard errors of intervention effect due to clustering of patient within centers (at the intercept and center levels). Missing data were assumed to represent cancellations for primary and secondary binary outcomes. All randomized patients (including erroneous inclusions) were included in the sensitivity analysis. Results were expressed as crude and adjusted odds ratios (95% CI). Significance was assumed at p<0.05 (two-tailed). All analyses used SAS software version 9.3 (SAS Inc, Cary, NC).

## Results

### Characteristics of the participants

**Fig 1:**
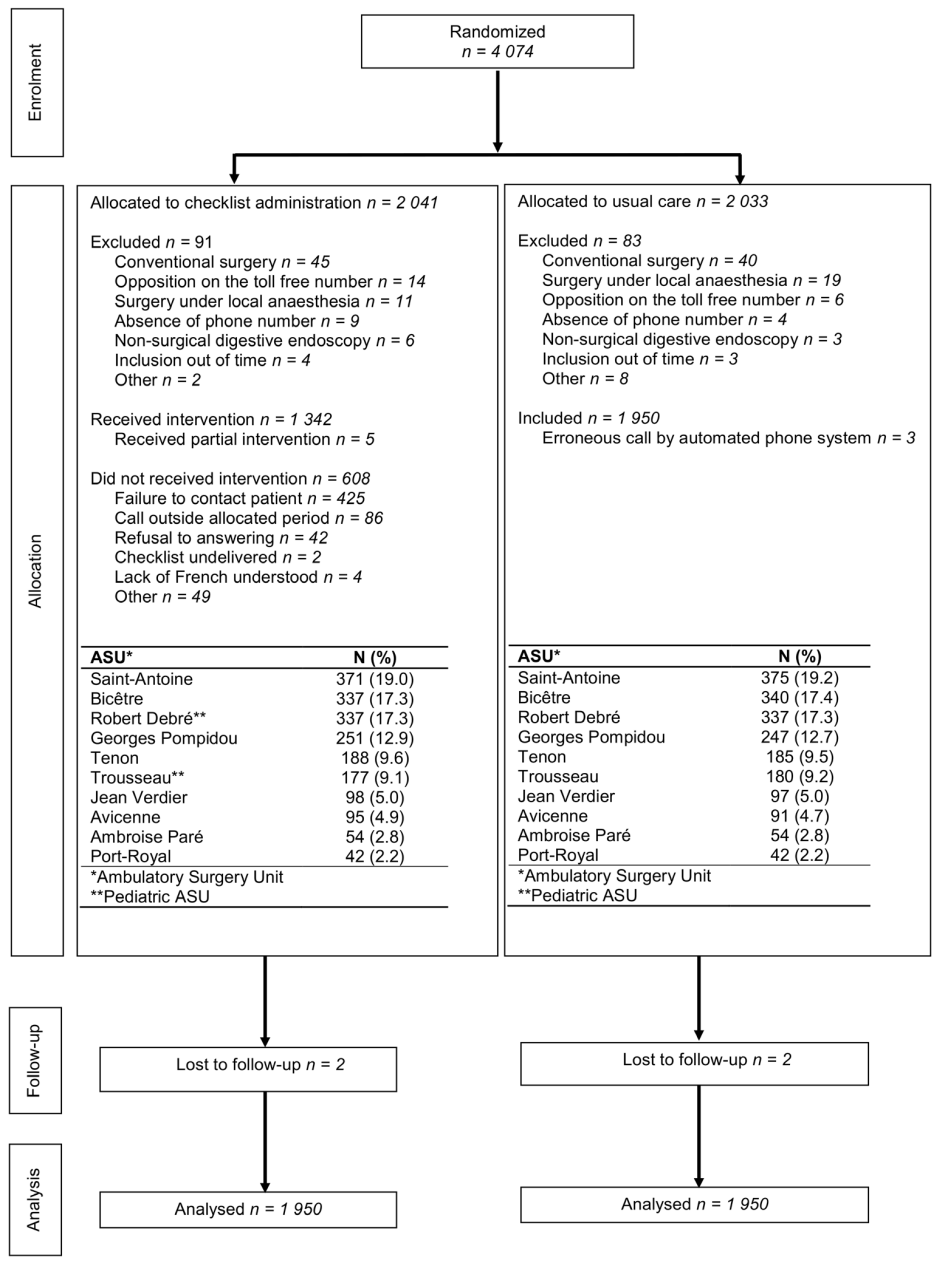
CONSORT diagram for the RCT AMBUPROG.

A total of 4074 patients were randomized between 21 November 2012 and 18 September 2013, of whom 2041 were allocated to the checklist arm and 2033 to the control arm. One hundred seventy-four patients (91 in the checklist arm, 83 in the control arm) were erroneously included, and 1950 patients were finally analyzed in each group. The rates of erroneous inclusion, and the reasons, were similar in the two groups. The individual ASUs contributed between 2.2% and 19.1% of the study population. A flow chart is provided in Fig 1. As shown in Table 2, 51.9% of patients were male; mean (SD) age was 34.2 (24.6) years; and mean BMI was 25.2 (5.2).

**Table 2:** Baseline characteristics of patients in the AMBUPROG trial.

Characteristics	AMBUPROG checklist group^a^ N = 1 950	Control arm^b^ N = 1 950
**Adults (age ≥ 18 years) N = 2565**		
Male sex, No. (%)	558 (43.5)	579 (45.1)
Female sex, No. (%)	724 (56.5)	704 (54.9)
Age, mean (SD) [range]	49.0 (16.9) [18.0–98.1]	48.8 (16.4) [18.0–90.6]
BMI, mean (SD) [range]	25.5 (5.2) [15.6–55.0]	25.6 (5.1) [15.4–49.2]
Diabetes, No. (%)	87 (7.1)	48 (3.9)
Coronary disease, No. (%)	57 (4.6)	44 (3.6)
Thromboembolism, No. (%)	44 (3.6)	88 (7.2)
**Children and Teenagers (age < 18 years) N = 1335**		
Male sex, No. (%)	442 (66.3)	444 (66.9)
Female sex, No. (%)	225 (33.7)	220 (33.1)
Age, mean (SD) [range]	5.8(4.4) [0.1–17.9]	6.0 (4.6) [0.1–17.9]
**ASA**^**c**^ **classification, No. (%)**		
ASA 1	1153 (62.1)	1132 (61.1)
ASA 2	635 (3.2)	643 (34.7)
ASA 3	63 (3.4)	77 (4.2)
ASA 4	5 (0.3)	2 (0.1)
**Surgical indications. No. (%)**		
Orthopedic	636 (32.6)	637 (32.7)
Visceral/digestive	400 (20.5)	456 (23.4)
ENT	243 (12.5)	217 (11.1)
Urology	240 (12.3)	222 (11.4)
Maxillofacial	148 (7.6)	129 (6.6)
Gynecologic	128 (6.6)	104 (5.3)
Plastic surgery	94 (4.8)	113 (5.8)
Thoracic	29 (1.5)	30 (1.5)
Oncology	13 (0.7)	28 (1.4)
Ophthalmology^d^	11 (0.6)	12 (0.6)
Endocrine	5 (0.3)	0 (0.0)
Cardiovascular	3 (0.2)	2 (0.1)

^a^ Missing data: BMI (n) and ASA (n) = 127 and 94, respectively.

^b^ Missing data: BMI (n) and ASA (n) = 133 and 96, respectively.

^c^ ASA: American Society of Anesthesiology.

^d^ Ophthalmologic indications were taken in account only in multidisciplinary USAs.

### Checklist administration

The checklist was delivered to a total of 1342 patients (68.8%), 1002 (74.7%) by the automated phone system and 340 (25.3%) by a research assistant. The checklist was not delivered to 608 patients (31.1%), because of failure to contact the patient (n = 425), call outside the allocated period (n = 86), or refusal to answer the checklist (n = 42).

The checklist revealed that 220 patients (17.3%) had not completed the necessary administrative procedures for their admission; that 125 patients (9.8%) had experienced a change in their health status since their last hospital consultation; that 355 patients (28.0%) had not had the tests requested by the surgeon or anesthetist; and that 254 patients (20.0%) had questions regarding the "empty stomach" instructions.

Detailed results are provided in Table 3.

**Table 3:** Results of 1342 administered checklists.

Items^a^	Type of response		No answer n (%)
	Yes n (%)	No n (%)	
**1: You are scheduled for ambulatory surgery in the next few days. Do you confirm the date of your surgery?**	1273 (94.9)	69 (5.1)	0 (0.0)
**2: Do you have an accompanying adult to take you home?**	1200 (94.3)	73 (5.7)	69 (5.1)
**3: Have you completed the administrative procedure for your admission?**	1051 (82.7)	220 (17.3)	71 (5.3)
**4: Has your health changed since your last hospital consultation, with a possible change in your regular treatment?**	125 (9.8)	1147 (90.2)	70 (5.2)
**5: Has the doctor or surgeon asked you to change your regular treatment in preparation for surgery?**	97 (7.7)	1166 (92.3)	79 (5.9)
**5b: If so, do you have any questions about the changes in your treatment?**	9 (9.3)	88 (90.7)	1245 (92.8)
**6: Has the doctor or surgeon asked you to undergo tests in preparation for surgery?**	355 (28.0)	912 (72.0)	75 (5.6)
**6b: If so, have you had the tests?**	317 (89.3)	38 (10.7)	987 (73.5)
**7: Do you have any questions about the instruction to stay on an empty stomach?**	254 (20.0)	1014 (80.0)	74 (5.5)

^a^ Items for adults are presented (the items for children are given in detail in Fig 1).

### Primary and secondary outcomes (Table 4)

There was no statistically significant difference between the two groups regarding the rate of late cancellation: 109 patients (5.6%) in the checklist arm and 113 patients (5.8%) in the control arm cancelled the day before surgery or on the day of surgery (adjusted odds ratio 0.91 (95%CI, 0.65; 1.29) (p = 0.57)).

**Table 4:** Primary and secondary endpoints (n, %).

	AMBUPROG checklist arm (N = 1950) n (%)	Control arm (N = 1950) n (%)	Crude OR (95%CI)	Adjusted OR (95%CI)	P value (adjusted)
**Late cancellation**^**a**^	109 (5.6)	113 (5.8)	0.96 (0.73;1.26)	0.91 (0.65;1.29)	0.57
**Cancellation the day before surgery**^**a**^	41 (2.1)	43 (2.2)	0.95 (0.62;1.47)	0.88 (0.47;1.65)	0.65
**Cancellation on the day of surgery**^**a**^	70 (3.6)	72 (3.7)	0.98 (0.70;1.36)	0.96 (0.68;1.36)	0.81
**Conventional hospitalization**	6 (0.3)	9 (0.5)	0.67 (0.24;1.87)	0.67 (0.23;1.96)	0.46

OR: Odds Ratio, 95%CI: 95% confidence interval.

^a^ 2 missing data per arm (imputed as cancellation).

Likewise, no statistically significant difference was found in any of the secondary endpoints:

- Cancellation the day before surgery: 41 patients (2.1%) in the checklist arm, 43 patients (2.2%) in the control arm (adjusted odds ratio 0.88 (95%CI, 0.47; 1.65) (p = 0.65));
- Cancellation on the day of surgery: 70 patients (3.6%) in checklist arm, 72 patients (3.7%) in the control arm (adjusted odds ratio 0.96 (95%CI, 0.68; 1.36) (p = 0.81)).
- Conversion to conventional hospitalization: 6 patients (0.3%) in checklist arm, 9 patients (0.5%) in the control arm (adjusted odds ratio 0.67 (95%CI, 0.23; 1.96) (p = 0.46)).

When all randomized patients were analyzed, the primary endpoint remained non-significant, with an adjusted odds ratio of 1.01 (95%CI, 0.82; 1.23) (p = 0.95).

### Adverse effects

None.

## Discussion

This is the first large multicenter randomized controlled trial of a standardized pre-operative checklist administered by an automated phone system with the aim of reducing late cancellations of ambulatory surgery. Use of the checklist did not reduce the rate of late cancellations but allowed us to identify several key factors that could lead to late cancellation. In particular, 28% of patients had not had the tests requested by the surgeon or anesthetist; 20% said they did not fully understand the instructions regarding the need to have an "empty stomach", and 17% had not completed the necessary pre-admission administrative formalities. However, identification of these issues through the checklist between day 7 and day 3 before the scheduled date of surgery had no impact on the rate of late cancellation by comparison with standard management. This suggests that standard management already in place in the ASUs (in particular, a standard phone call to the patient the day before surgery) was able to address these issues. Another explanation could be that other reasons for late cancellation were not addressed by our generic checklist. Indeed, patient-specific reasons could have contributed to the rate of late cancellation in both groups. Our study was not tailored to identify patient-specific reasons for late cancellation and further studies are therefore needed to address these issues.

This study has several strengths. First, the randomized controlled trial design and the small proportion of missing data minimizes the selection bias. Second, the study was particularly large, with more than 4000 patients recruited in eleven multidisciplinary ASUs that were representative in terms of their volume of activity, patient recruitment and surgical specialties. Third, the intervention (checklist) was developed through multidisciplinary collaboration among surgeons, anesthetists and nurses working in 12 different surgical specialties. Finally, the checklist was tailored to the patients’ characteristics (adults, children) and geographic origin (5 different languages).

Several studies of interventions designed to reduce late cancellations have been already published [13–23]. Three of them concerned ambulatory surgery [13,15,21]. The interventions consisted of pre-operative medical assessment [14,19–20], telephone interviews [13,15–16,21], nurse-led pre-assessment [17], SMS text messaging [18], or telephone calls [22,23]. Pre-operative patient assessment a few days or weeks before the scheduled date of surgery appeared to reduce the risk of cancellation. However, none of these studies was randomized, and the results might therefore have been subject to a selection bias.

The lack of impact of our checklist is puzzling. It is conceivable that the checklist was not sufficiently tailored to the specific causes of late cancelation in the 11 ASUs, or to the patient population. Indeed, to allow its general use, the checklist was very broad and generic. Furthermore, it was based on patient self-management in order to avoid excess costs and an extra workload for the ASU staff. A checklist customized to individual patients and their surgical indications might be more effective. Second, uptake of the checklist was poor, possibly because some patients were put off by the automated phone system. Indeed, the checklist had to be delivered by a research assistant to nearly one-quarter of patients. It also proved difficult to reach the patients by telephone. Thus, among the 1950 patients allocated to the checklist arm, more than one-third did not complete the checklist (n = 608), mainly because they could not be contacted (n = 425). Finally, the impact of the checklist might have been limited by the pre-existing procedure used in most of the ASUs, based on a routine phone reminder the day before surgery.

Our study also has some limitations. A total of 174 patients were erroneously included (91 patients in the checklist arm, 83 patients in control arm). However, a sensitivity analysis including these patients yielded the same results. Also, more than one-third of patients did not receive the intervention, although this is likely representative of our clinical practice.

In conclusion, the AMBUPROG pre-surgical checklist delivered by an automated phone system did not reduce the rate of late cancellation of ambulatory surgery. This study is none the less encouraging, not least because it demonstrates the feasibility of a large-scale randomized controlled trial of this type in 11 ASUs with very different working practices. Further studies are needed to assess more personalized pre-surgical checklists.

## Supporting Information

S1 CONSORT Checklist CONSORT Checklist. (PDF)

S1 Protocol Original study protocol. (PDF)

S2 Protocol Supplementary data. (PDF)

S3 Protocol Supplementary data. (PDF)

S4 Protocol Supplementary data. (PDF)

S5 Protocol Supplementary data. (PDF)

S6 Protocol Supplementary data. (PDF)

S7 Protocol Supplementary data. (PDF)

S8 Protocol Supplementary data. (PDF)
