# Supplementary material for: Assessment of a Standardized Pre-Operative Telephone Checklist Designed to Avoid Late Cancellation of Ambulatory Surgery: The AMBUPROG Multicenter Randomized Controlled Trial
Source: PLoS One. 2016 Feb 1;11(2):e0147194. doi: 10.1371/journal.pone.0147194 (PMC4734771; doi:10.1371/journal.pone.0147194)
Supplement: S1 Protocol — (PDF) [file pone.0147194.s002.pdf]

ASSISTANCE PUBLIQUE HÔPITAUX DE PARIS

AMBUPROG

Impact d'une « check-list » sur le nombre de déprogrammations tardives des patients en chirurgie ambulatoire

PROTOCOLE DE RECHERCHE VISANT A EVALUER LES SOINS COURANTS

Version N°4-0 du 20/06/2013
Codes projet : **PHRQ1145 / K110601 / N°ID RCB : 2011-A01647-34**

Investigateur coordonnateur :

Pr Jean-Pierre BETHOUX
Service de Chirurgie Générale, Plastique et Ambulatoire
Hôpitaux Universitaires Paris Centre
Nouveau Bâtiment Port-Royal
53 Avenue de l'Observatoire
75014 Paris

☏ : 01 58 41 37 82-

E-mail : jean-pierre.bethoux@cch.aphp.fr

Gestionnaire :

AP-HP- DRCD
1, avenue Claude Vellefaux
75010 PARIS
Référent projet : Ludovic DYEN
☏ : 01.44.84.17.43 - 🖷 : 01.44.84.17.01

E-mail : ludovic.dyen@sls.aphp.fr

URC Cochin-Necker
Référent projet : Sabine HELFEN
GH Cochin – site Tarnier

89 rue d'Assas – 75006 Paris
☏ : 01.58.41.11.90 - 🖷 : 01.58.41.11.83

E-mail : sabine.helfen@cch.aphp.fr

Département de la Recherche Clinique et du Développement
DIRC Ile de France
Hôpital Saint Louis 75010 PARIS

Page de SIGNATURE D'UN PROTOCOLE de recherche visant à évaluer les soins courants

Codes de la Recherche : **PHRQ1145 / K110601 / N°ID RCB : 2011-A01647-34**

Titre : **AMBUPROG : Impact d'une « check-list » sur le nombre de déprogrammations tardives des patients en chirurgie ambulatoire**

Version N°4-0 du 20/06/2013

La recherche sera conduite conformément au protocole et aux dispositions législatives et réglementaires en vigueur.

L'investigateur coordonnateur :

Pr Jean-Pierre BETHOUX
Service de Chirurgie Générale, Plastique et Ambulatoire
Hôpitaux universitaires Paris Centre
Nouveau Bâtiment Port-Royal
53 Avenue de l'Observatoire
75014 Paris

Date : ……………/………/………..
Signature :

Le responsable scientifique :

Dr Isabelle BOUTRON
Centre d'épidémiologie Clinique INSERM U738
Hôpital Hôtel Dieu
1, place du Parvis Notre-Dame
75181 Paris cedex 4

Date : ……………/………/………..
Signature :

Le gestionnaire :

Christophe MISSE
Directeur du DRCD
Assistance Publique – Hôpitaux de Paris
Département de la Recherche Clinique et du Développement
Hôpital Saint Louis
75010 PARIS

Date : ……………/………/………..
Signature :

La recherche a reçu un avis favorable initial du CPP IDF I, Hôtel Dieu en date du 30/01/2012, l'avis favorable pour la MS 1 23/07/2012, l'avis favorable pour la MS 2 le 12/02/2013 et l'avis favorable pour la MS 3 le 27/06/2013.

TABLE DES MATIÈRES

1. RESUME SYNOPTIQUE 3

2. RATIONNEL DE LA RECHERCHE 3

2.1. ETAT ACTUEL DES CONNAISSANCES AU REGARD DE LA RECHERCHE 3
2.2. QUALIFICATION DE LA RECHERCHE 3

3. OBJECTIFS DE LA RECHERCHE 3

3.1. HYPOTHESES DE LA RECHERCHE 3
3.2. OBJECTIF PRINCIPAL DE LA RECHERCHE 3

4. ACTES ET STRATEGIES MEDICALES DE PRATIQUE COURANTE EVALUES PAR LA RECHERCHE 3

4.1. ELABORATION DE LA « CHECK-LIST » 3
4.2. MODE D'ADMINISTRATION DE LA « CHECK-LIST » 3

5. CONCEPTION DE LA RECHERCHE 3

5.1. METHODOLOGIE DE LA RECHERCHE 3
5.2. NOMBRE DE CENTRES PARTICIPANTS PREVUS 3
5.3. RECHERCHE NATIONALE/INTERNATIONALE 3
5.4. TIRAGE AU SORT 3
5.5. CRITERES D'EVALUATION PRINCIPAL ET SECONDAIRES 3

6. DEROULEMENT DE LA RECHERCHE 3

6.1. CALENDRIER DE LA RECHERCHE 3
6.2. VISITE D'INCLUSION ET RANDOMISATION D'UN PATIENT 3
6.3. ADMINISTRATION DE LA CHECK-LIST ET SUIVI DE LA RECHERCHE 3
6.4. VISITE DE FIN DE LA RECHERCHE 3
6.5. TABLEAU OU SCHEMA RECAPITULATIF DE LA CHRONOLOGIE DE LA RECHERCHE 3

TABLEAU 2 : TABLEAU RECAPITULATIF DE LA CHRONOLOGIE DE LA RECHERCHE 3

6.6. REGLES D'ARRET DEFINITIF OU TEMPORAIRE DE LA RECHERCHE 3

ARRET D'UNE PARTIE OU DE LA TOTALITE DE LA RECHERCHE : SI LE TAUX DE RECRUTEMENT N'EST PAS ATTEINT, LA RECHERCHE SERA STOPPEE 3

7. SELECTION DES PERSONNES 3

7.1. CRITERES D'INCLUSION 3
7.2. CRITERES DE NON INCLUSION 3

8. MODALITES DE RECRUTEMENT DES PERSONNES 3

9. MODALITES D'INFORMATION DES PERSONNES 3

10. GESTION DES EVENEMENTS INDESIRABLES 3

10.1. EVENEMENTS INDESIRABLES (I.E. COMPLICATIONS) LIES AUX ACTES, COMBINAISONS D'ACTES OU STRATEGIES MEDICALES DE PREVENTION, DE DIAGNOSTIC OU DE TRAITEMENT QUI SONT DE PRATIQUE COURANTE (HORS MEDICAMENT) DU SOIN 3

11. GESTION DES DONNEES 3

11.1. DROIT D'ACCES AUX DONNEES ET DOCUMENTS SOURCE 3
11.2. CONTROLE ET ASSURANCE DE LA QUALITE 3

TOUTE VISITE FERA L'OBJET D'UN RAPPORT DE VISITE PAR COMPTE-RENDU ECRIT. 3

11.3. TRAITEMENT DES DONNEES ET CONSERVATION DES DOCUMENTS ET DES DONNEES RELATIVES A LA RECHERCHE 3

12. ASPECTS STATISTIQUES 3

12.1. JUSTIFICATION DE LA TAILLE DE L'ECHANTILLON 3

12.2. DESCRIPTION DES METHODES STATISTIQUES PREVUES Y COMPRIS DU CALENDRIER DES ANALYSES INTERMEDIAIRES PREVUES 3

13. ASPECTS ETHIQUES ET LEGAUX **3**

13.1. DECLARATION INDIQUANT QUE LA RECHERCHE SERA CONDUITE CONFORMEMENT AU PROTOCOLE, AUX DISPOSITIONS LEGISLATIVES ET REGLEMENTAIRES EN VIGUEUR........ 3

LA RECHERCHE SERA CONDUITE CONFORMEMENT AU PRESENT PROTOCOLE. **3**

13.2. EVALUATION ETHIQUE DES MODALITES PARTICULIERES DE SURVEILLANCE PREVUES PAR LE PROTOCOLE........ 3

13.3. OBLIGATIONS LEGALES (ROLE DU GESTIONNAIRE, CPP, CCTIRS, CNIL) 3

IL SOUMET LE DOSSIER A L'AVIS DU COMITE DE PROTECTION DES PERSONNES CONCERNE. **3**

14. BIBLIOGRAPHIE **3**

15. ANNEXES **3**

ANNEXE 1........ **3**

ANNEXE 2........ **3**

CHECK-LIST **3**

1. RESUME SYNOPTIQUE

**Titre**	**AMBUPROG**<br>Impact d'une « check-list » sur le nombre de déprogrammations tardives des patients en chirurgie ambulatoire.
**Version du protocole**	**version 4.0 du 20/06/2013**
**Source de financement**	Lauréat de l'appel d'offre PREQHOS
**Gestionnaire**	Assistance Publique – Hôpitaux de Paris (AP-HP)
**Investigateur Coordonnateur**	Pr Jean-Pierre BETHOUX
**Nature du soin courant évalué**	Impact d'une check-list administrée aux patients sur les déprogrammations tardives en chirurgie ambulatoire
**Population concernée**	Tous les patients majeurs et mineurs programmés pour une chirurgie ambulatoire sauf critères d'exclusion
**Nombre de centres prévus**	11 centres hospitaliers de l'AP-HP
**Nombre de patients prévus**	4090 avec<br>- Groupe contrôle : 2045<br>- Groupe avec « check-list » : 2045
**Objectif principal**	Evaluation de l'impact d'une « check-list » sur le nombre de déprogrammations tardives (la veille ou le jour de l'intervention) des patients en chirurgie ambulatoire
**Critères d'inclusion**	1. Patient (adulte ou mineur) pour lequel une intervention chirurgicale ambulatoire est programmée dans une UCA polyvalente<br>2. Chirurgie sous anesthésie générale ou loco-régionale ou neuroleptanalgésie<br>3. Patient joignable par téléphone<br>4. Patient ne s'opposant pas à sa participation dans l'étude
**Critères de non inclusion**	1. Patient programmé en UCA pour une endoscopie non chirurgicale<br>2. Chirurgie ambulatoire programmée pour une orthogénie<br>3. Chirurgie ambulatoire programmée en urgence<br>4. Chirurgie ambulatoire réalisée sous anesthésie locale
**Critères d'évaluation**	- Le nombre de déprogrammation des patients en chirurgie ambulatoire la veille ou le jour de l'admission (J-1 ou J0)<br>- Le nombre de déprogrammation des patients en chirurgie ambulatoire à J-1<br>- Le nombre de déprogrammation des patients en chirurgie ambulatoire à J0<br>- Le nombre de passage en hospitalisation conventionnelle
**Méthodologie de la recherche**	Etude nationale de soins courants, multicentrique, randomisée en ouvert
**Durée de la recherche**	- Durée de la période d'inclusion : 33 mois<br>- Durée de participation du patient : inférieure ou égal à 1 mois<br>- Durée totale de la recherche : 3 ans

2. RATIONNEL DE LA RECHERCHE

2.1. ETAT ACTUEL DES CONNAISSANCES AU REGARD DE LA RECHERCHE

2.1.1. Sur les stratégies/procédures de référence

La chirurgie ambulatoire accuse un retard important dans notre pays. Au-delà de ce constat, la priorité est à l'analyse des causes et à la mise en place des moyens potentiellement efficaces pour favoriser son développement. De nombreux paramètres faisant intervenir des impératifs financiers, médicaux et logistiques sont à prendre en compte. Parmi ces paramètres, l'aspect organisationnel, centré sur la notion de « parcours patient » (« clinical pathway » des Anglo-Saxons) occupe une place prépondérante [1, 2].

Le séquençage des différentes étapes de la prise en charge des patients devant bénéficier d'une chirurgie en ambulatoire, de la consultation de chirurgie jusqu'à l'appel au domicile le lendemain de l'intervention, permet l'identification de certains problèmes. Il en est ainsi du taux d'annulation au dernier moment, qui est un paramètre global largement reconnu comme un indice d'efficience et de la qualité des soins. Toutes les études sur de grandes cohortes, mêlant différents types de chirurgies rapportent un taux d'annulation au dernier moment entre 7 et 13% des cas[3, 4]. Parmi les raisons les plus souvent retrouvées, on peut isoler celles relatives aux patients (décision de surseoir à l'intervention, oubli, maladie intercurrente, mauvais respect des consignes préopératoires…), celles liées à l'évaluation médicale préopératoire (absence d'un délai suffisant pour réaliser un examen, mauvaise explication des consignes…), et enfin celles purement attribuables à la structure (absence du chirurgien, défaut de programmation, manque du matériel chirurgical, défaut de transfert d'informations….)[3, 4].

Le taux d'annulation est un événement important, tant dans ses répercussions, que dans sa signification[5]. Les conséquences financières de l'annulation au dernier moment sont importantes, liées à la sous-occupation et à la désorganisation de l'activité des blocs opératoires[3]. Il existe également des conséquences directes sur la motivation des équipes et sur la satisfaction des patients. Enfin, un taux d'annulation élevé reflète des dysfonctionnements dans l'organisation de la structure de soin.

La mise au point de procédures visant à détecter, prévenir et à analyser les causes d'annulation en ambulatoire est un enjeu majeur pour les unités d'ambulatoire[1, 2]. L'emploi d'un outil informatique de type « Check list », réalisée systématiquement en période préopératoire, pourrait apporter une solution à ce problème. Ainsi, en plus de son rôle de détection préopératoire des risques d'annulation liés aux patients, cette « Check List » permettrait d'éviter certaines situations d'annulations pour causes médicales ou organisationnelles[6].

2.2. QUALIFICATION DE LA RECHERCHE

Ce protocole s'inscrit dans le cadre d'une recherche visant à évaluer les soins courants telle que définie par la loi n°2004-806 du 9 août 2004 relative à la politique de santé publique et par son décret d'application (n° 2006-477) du 26 avril 2006. (Textes de référence : articles L.1121-1, 2e alinéa et R1121-3 du Code de la santé publique).

2.2.1. Eléments démontrant que les stratégies médicales objets de la recherche, les actes pratiques et les méthodes utilisées au cours de la recherche sont conformes à la pratique courante

Le comparateur est la prise en charge usuelle telle qu'elle est organisée par chaque centre. Une enquête auprès des 11 unités de chirurgie ambulatoire (UCA) participant, prenant en charge des adultes ou des enfants, a été réalisée afin de décrire cette prise en charge usuelle au début de l'étude. Ainsi, actuellement, 9 UCA sur les 11 utilisent une check-list administrée généralement à J-1 par téléphone sans assurance sur l'aboutissement de cette démarche.

2.2.2. Eléments justifiant que les modalités particulières de surveillance ajoutées par la recherche ne comportent que des risques et contraintes négligeables

**Modalités de surveillance réalisées dans le cadre du soin** (Prise en charge habituelle)	**Modalités particulières de surveillance ajoutées par la recherche** (Actes supplémentaires par rapport à la prise en charge habituelle)
Les centres appliquent, pour la plupart, une check-list.<br>Les check-lists utilisées localement sont différentes d'un centre à l'autre<br>La période d'administration de cette check-list est différente d'un centre à l'autre (entre J-3 et J-1)	*Une check-list commune est administrée dans les 11 UCA participant.<br>L'administration de cette check-list est plus en amont de la chirurgie : entre J-7 et J-3.<br>Cette check-list commune est administrée en parallèle avec la check list habituelle propre à chaque UCA*

Tableau 1 : Modalités particulières de surveillance réalisées dans le cadre du soin / ajoutées par la recherche

Le seul acte supplémentaire ajouté par la recherche est un appel téléphonique au patient, ou aux parents lorsqu'il s'agit d'un mineur, qui doit se faire prochainement opéré.

3. OBJECTIFS DE LA RECHERCHE

3.1. HYPOTHESES DE LA RECHERCHE

L'utilisation d'une « check-list » a déjà démontré son intérêt sur l'amélioration de la qualité des soins dans d'autres circonstances [7-10, 11, 12-16]
Une condition essentielle du succès de la chirurgie ambulatoire repose sur l'organisation logistique du flux des patients. Actuellement, l'organisation des hospitalisations en chirurgie ambulatoire souffre d'un nombre non négligeable de déprogrammations tardives c'est-à-dire la veille ou le jour même de l'intervention. Ces déprogrammations tardives ont un coût important car il n'est plus possible, dans les délais, de réorganiser le programme opératoire. On estime qu'actuellement dans les services de l'APHP qui participeront à cette étude, le taux de déprogrammation tardive (la veille ou le jour de l'intervention) concerne 10% des séjours.
Il parait par conséquent essentiel de limiter ce taux de déprogrammations tardives et notre hypothèse repose sur le fait que l'administration d'une « check-list » à tous les patients programmés pour une intervention de chirurgie ambulatoire à distance de la date d'hospitalisation pourrait être un moyen efficace de limiter ces déprogrammations tardives.

Conséquences attendues des résultats du projet :
Les résultats de cette étude auront un impact important pour l'organisation de la chirurgie ambulatoire. En effet, si l'on démontre que l'administration d'une check-list permet de diminuer le taux de déprogrammation et ainsi d'éviter une désorganisation importante et des coûts inutiles, on pourrait dans un premier temps l'utiliser en routine à l'APHP puis proposer d'étendre l'utilisation de cette check-list.
Par ailleurs, cette étude sera une opportunité d'homogénéiser les pratiques dans les différents centres de chirurgie ambulatoire impliqués.
L'analyse des causes d'annulation permettra également la mise en place de mesures correctives aptes à améliorer le fonctionnement des unités de chirurgie ambulatoire.

3.2. OBJECTIF PRINCIPAL DE LA RECHERCHE

L'objectif principal de cette étude est d'évaluer l'impact d'une « check-list » par rapport à une prise en charge usuelle sur le nombre de déprogrammations tardives (la veille ou le jour de l'intervention) des patients en chirurgie ambulatoire.

4. ACTES ET STRATEGIES MEDICALES DE PRATIQUE COURANTE EVALUES PAR LA RECHERCHE

Il s'agit d'évaluer l'impact d'une check-list standardisée, administrée au patient sur une période comprise entre 7 jours (J-7) et 3 jours (J-3) avant son intervention chirurgicale.

4.1. Elaboration de la « check-list »

La « check-list » a été élaborée en 2 étapes. Dans un premier temps, il a été demandé à tous les investigateurs de proposer des items (sans limite de nombre) qui pourraient être utilisés pour la « check-list ». Les investigateurs pouvaient faire intervenir des membres de leur équipe médicale et paramédicale. A l'issue de cette première étape, une réunion de discussion a été organisée. Au cours de cette réunion, une liste d'items a été proposée avec des modalités d'action en fonction des réponses des patients. Cette liste d'items préliminaire a été diffusée à l'ensemble des participants qui ont proposé des modifications notamment de formulation des items. Finalement cette « check-list » a été testée sur un panel de soignants et de patients volontaires. Les remarques des patients ont été prises en compte et ont permis de modifier la check-list.

4.2. Mode d'administration de la « check-list »

Les coordonnées téléphoniques et le numéro d'inclusion (mais pas l'identité complète) de tous les patients randomisés « avec administration de la check-list » seront transmises à un centre d'appel pour programmer l'appel. La « check-list » sera systématiquement administrée aux patients par téléphone, en cinq langues au choix (français, anglais, portugais, chinois mandarin ou arabe du Maghreb) par un système de serveur vocal. Si les patients ne souhaitent pas répondre au système vocal, ils seront contactés par téléphone par des techniciens de recherche clinique (TEC). En fonction des réponses des patients aux questions de la check-list, un message électronique sera, le cas échéant, adressé au référent du service concerné. D'autre part, le TEC/Système vocal pourra demander au patient de contacter l'UCA concernée. Chaque service, en fonction de son organisation interne, mettra en place des mesures correctrices: appel du patients pour corriger le problème ou pour annuler / reporter l'intervention. La check-list est disponible en Annexe 2.
Cette check-list sera administrée en plus de la prise en charge usuelle de chaque centre. Il est à noter que de nombreux centres contactent les patients par téléphone la veille de l'intervention.

5. CONCEPTION DE LA RECHERCHE

5.1. METHODOLOGIE DE LA RECHERCHE

Type de recherche :

- essai contrôlé
- randomisée par patient
- en 2 groupes parallèles

5.2. NOMBRE DE CENTRES PARTICIPANTS PREVUS

Onze UCA polyvalents de l'Assistance Publique des Hôpitaux de Paris sont impliqués dans cette étude.

5.3. RECHERCHE NATIONALE/INTERNATIONALE

La recherche est nationale

5.4. TIRAGE AU SORT

- la nécessité de tirage au sort : L'essai contrôlé randomisé est la méthode de référence pour l'évaluation thérapeutique. La randomisation permettra de limiter les biais de sélection et de permettre une estimation non biaisée des résultats.

-la méthode de tirage au sort : La randomisation 1 :1 sera centralisée et mise en application via le cahier d'observation électronique

-le type de randomisation (patient ou cluster) : Une liste de randomisation générée par ordinateur avec des blocs de taille variable sera établie par un statisticien. Les médecins n'auront pas accès à cette liste de randomisation. La randomisation sera stratifiée sur le centre.

5.5. CRITERES D'EVALUATION PRINCIPAL ET SECONDAIRES

5.5.1. Critère d'évaluation principal

Le critère de jugement principal sera le nombre de déprogrammations des patients en chirurgie ambulatoire la veille ou le jour de l'admission (J-1 ou J0).
L'information de la déprogrammation est une donnée administrative systématiquement collectée dans le cadre du soin. Elle sera recueillie dans le cahier d'observation de l'étude.

5.5.2. Critères d'évaluation secondaires

Les critères de jugement secondaires seront :

- Le nombre de déprogrammation des patients en chirurgie ambulatoire à J-1
- Le nombre de déprogrammation des patients en chirurgie ambulatoire à J0
- Le nombre de passage en hospitalisation conventionnelle

Ces informations sont des données administratives systématiquement collectées dans le cadre du soin. Elles seront recueillies dans le cahier d'observation de l'étude.

6. DEROULEMENT DE LA RECHERCHE

L'étude se déroulera de la manière suivante :

6.1. Calendrier de la recherche

- Durée de la période d'inclusion : 33 mois

- Durée de participation de chaque patient : inférieure ou égale à 1 mois : le patient sera inclus dès programmation de son opération jusqu'à obtention de ses réponses à la check-list. (La durée de validité d'une consultation d'anesthésie générale est de 2 mois)

- Durée totale de la recherche : 3 ans

- Le moment de la réalisation de la randomisation *:* le patient sera randomisé entre le jour de la programmation de son opération (J0) et jusqu'à 4 jours avant J0

6.2. Visite d'inclusion et randomisation d'un patient

La visite d'inclusion a lieu entre *30 jours* et au plus tard *7 jours* avant la chirurgie.
Cette visite se définie par la remise au patient de la note d'information de cette recherche lors de son parcours de consultations visant à organiser son intervention chirurgicale. La remise de l'information peut être réalisée soit le jour de la programmation de l'intervention, soit le jour de la consultation d'anesthésie

Après diffusion du programme opératoire chirurgical dans chaque centre, et après vérification de la non-opposition du patient, les données démographiques générales ainsi que la date de l'intervention des patients seront collectées dans un e-CRF. Le patient sera identifié par un numéro d'inclusion dans l'ordre d'arrivée dans la recherche et ses initiales.

Les patients seront ensuite randomisés par un module informatisé intégré au cahier électronique.

6.3. Administration de la check-list et suivi de la recherche

Il n'y a aucune visite de consultation à proprement parlé pour cette recherche

Les patients randomisés dans le bras prise en charge usuelle auront la prise en charge habituelle proposée dans le centre. Les coordonnées téléphoniques de tous les patients randomisés « avec administration de la check-list » seront transmises à un centre d'appel pour programmer l'appel. La « check-list » sera systématiquement administrée aux patients par téléphone, en cinq langues au choix (français, anglais, portugais, chinois mandarin ou arabe du Maghreb par un système vocal. Si les patients ne souhaitent pas répondre au système vocal, ils seront contactés par téléphone par des techniciens de recherche clinique (TEC). En fonction des réponses des patients aux questions de la check-list, un message électronique sera ,le cas échéant, adressé au référent du service concerné. D'autre part, le TEC/Système vocal pourra demander au patient de contacter l'UCA concernée. Chaque service, en fonction de son organisation interne, mettra en place des mesures correctrices: appel du patients pour corriger le problème ou pour annuler / reporter l'intervention. La check-list est disponible en Annexe 2.

Cette check-list sera administrée en plus de la prise en charge usuelle de chaque centre. Il est à noter que 9 UCA contactent les patients par téléphone la veille de l'intervention.

6.4. Visite de fin de la recherche

La participation du patient prendra fin le jour de son intervention chirurgicale. Aucun examen ne sera réalisé pour cette recherche lors de l'intervention.

6.5. Tableau ou schéma récapitulatif de la chronologie de la recherche

	inclusion<br>j-30 à j-4	Randomisation<br>j-7 à j-3	j-7 à j-3	J0
Information	✓			
Non opposition	✓		✓	
Diffusion du programme opératoire		✓		
Administration de la check-list			✓	
Intervention chirurgicale ambulatoire				✓

Tableau 2 : Tableau récapitulatif de la chronologie de la recherche

6.6. Règles d'arrêt définitif ou temporaire de la recherche

Les patients pourront sortir de l'étude dans les conditions suivantes :

Arrêt de la participation d'une personne à la recherche :

- si la personne signifie son opposition

- si les conditions de l'intervention chirurgicale sont modifiées et qu'un critère de non-inclusion apparaît avant sa randomisation

Arrêt d'une partie ou de la totalité de la recherche : Si le taux de recrutement n'est pas atteint, la recherche sera stoppée

Modalités et calendrier de recueil pour ces données : aucune donnée ne sera recueillie pour les patients ayant signifié leur opposition.

Les données des patients randomisés seront recueillies jusqu'au jour de l'intervention et seront analysées

Modalités de suivi de ces personnes : Pas de suivi dans le cadre de cette recherche

7. SELECTION DES PERSONNES

7.1. CRITERES D'INCLUSION

1) Patient (adulte ou mineur) pour lequel une intervention chirurgicale ambulatoire est programmée dans une UCA polyvalente
2) Chirurgie sous anesthésie générale ou loco-régionale ou neuroleptanalgésie
3) Patient joignable par téléphone
4) Patient ne s'opposant pas à sa participation dans l'étude

7.2. CRITERES DE NON INCLUSION

1) patient programmé dans une UCA participante pour une endoscopie non chirurgicale
2) Chirurgie ambulatoire programmée pour une orthogénie
3) Chirurgie ambulatoire programmée en urgence
4) Chirurgie ambulatoire programmée sous anesthésie locale

8. MODALITES DE RECRUTEMENT DES PERSONNES

Estimation du potentiel d'inclusion par an et par centre après l'enquête de faisabilité réalisée préalablement dans tous les centres participants :

**Centres**	**Potentiel d'inclusion / 10 mois**
Centre 1	700 patients
Centre 2	3000
Centre 3	600
Centre 4	500
Centre 5	1600
Centre 6	1700
Centre 7	1400
Centre 8	2200
Centre 9	1500
Centre 10	500
Centre 11	400
Total	14100 patients

Tableau 3 : Potentiel d'inclusion

9. MODALITES D’INFORMATION DES PERSONNES

Conformément à **la loi n°2004-806 du 9 août 2004 relative à la politi que de santé publique**, l’investigateur a **l’obligation d’informer les personnes** préalablement à leur participation à une recherche visant à évaluer les soins courants.

9.1.1. Information de la personne

Conformément à l’article R. 1121-3 du Code de la santé publique, l'information des personnes qui se prêtent à la recherche fait l'objet d'un document écrit soumis préalablement au comité de protection des personnes intéressé.

Tous les patients, ou le ou les titulaires de l’autorité parentale lorsqu’il s’agit d’un mineur, pour lesquels une intervention chirurgicale ambulatoire est prévue seront informés de cette étude. Tous les patients répondants aux critères d’inclusion, inscrits sur le programme opératoire et n’ayant pas émis d’opposition à leur participation à cette étude seront inclus dans cette étude.
Les patients mineurs n’ont pas de note d’information dédiée car la check-list sera administrée au titulaire de l’autorité parentale.
Une note d’information sera remise en main propre au moment de la consultation de chirurgie, d’anesthésie ou de l’inscription dans l’UCA en fonction des habitudes de chaque centre. La note d’information précise les objectifs de l’étude, la méthodologie utilisée et invite les patients à contacter un numéro de téléphone gratuit spécifiquement dédié en cas d’opposition à leur participation à cette étude. Lors de cet appel le patient va s’identifier (nom, prénom, date et lieu de la chirurgie), et le refus sera traité directement par le TEC de l’étude. Ce numéro de téléphone sera disponible 24 heures sur 24, 7 jours sur 7 et est indépendant du serveur vocal.
Le TEC vérifiera systématiquement, avant tout enregistrement de l’inclusion, si le patient n’a pas signifié son refus de participer. En cas de refus le patient ne sera ni inclus ni randomisé.
Par ailleurs des affiches expliquant l’étude et indiquant aux patients leur droit à s’opposer à leur participation seront systématiquement disposées dans les salles d’attente de consultation de chirurgie et d’anesthésie, et des secrétariats/salles d’attente UCA.
Une notification que l’information a été donnée aux patients sera retrouvée dans les centres participants.

Lorsque cette recherche sera terminée, la personne qui se prête à la recherche pourra être informée des résultats globaux de cette recherche selon les modalités qui lui seront précisées dans le document d'information.

9.1.2. (Non) opposition de la personne à la participation à la recherche

Dans le cadre des recherches visant à évaluer les soins courants, la personne, l’autorité parentale a la faculté de s’opposer à sa participation à la recherche. Ceci est précisé dans le document d’information prévu à l’article R1121-3 du Code de la

santé publique. (Cf. *Arrêté du 9 mars 2007 fixant la composition du dossier de demande d'avis au comité de protection des personnes pour les recherches visant à évaluer les soins courants mentionnées au 2° de l'a rticle L. 1121-1 du code de la santé publique)*

L'information donnée à la personne et son absence d'opposition doivent être notifiées et datées dans son dossier médical. Si la personne s'oppose à son inclusion dans la recherche, elle continuera à bénéficier de sa prise en charge habituelle.

10. GESTION DES EVENEMENTS INDESIRABLES

10.1. EVENEMENTS INDESIRABLES (I.E. COMPLICATIONS) LIES AUX ACTES, COMBINAISONS D'ACTES OU STRATEGIES MEDICALES DE PREVENTION, DE DIAGNOSTIC OU DE TRAITEMENT QUI SONT DE PRATIQUE COURANTE (HORS MEDICAMENT) DU SOIN

Dans le cadre des recherches visant à évaluer les soins courants, les actes ou les stratégies médicales, objets de la recherche, font partie de la pratique habituelle et sont utilisés dans le respect de leurs indications. Les événements indésirables potentiels sont donc ceux liés à la prise en charge habituelle du patient (liés au soin) et ne requièrent pas de déclaration spécifique de la part du gestionnaire de la recherche.

10.1.1. Evénements indésirables (i.e. complications) liés aux soins évalués dans le cadre de la recherche

Aucun risque n'est ajouté à cette étude et aucun événement indésirable n'est attendu en lien avec l'administration de la check-list.

Aucun événement ne sera notifié dans le cadre de cette recherche.

11. GESTION DES DONNEES

11.1. DROIT D'ACCES AUX DONNEES ET DOCUMENTS SOURCE

11.1.1. Accès aux données et confidentialité

Le gestionnaire s'assure que le protocole ainsi que la lettre d'information destinée aux personnes incluses dans la recherche et au représentant légal d'un mineur, précise que les investigateurs mettent les documents et données individuelles strictement nécessaires au suivi, au contrôle de qualité et à l'audit d'une recherche visant à évaluer les soins courants à la disposition des personnes individuellement mandatées à cet effet par le gestionnaire de la recherche. Le gestionnaire s'assure également que chaque personne qui se prête à la recherche ne s'est pas opposée à l'accès aux données individuelles le concernant.

Ces activités doivent obéir à des règles strictes en matière de confidentialité. Les personnes ainsi mandatées sont soumises au secret professionnel, notamment dans les conditions définies par les articles 226-13 et 226-14 du Code pénal, au même titre que les investigateurs eux-mêmes.

Pendant la recherche et à son issue, les données recueillies sur les personnes qui s'y prêtent et transmises au gestionnaire par les investigateurs (ou tous autres collaborateurs à la recherche) seront codifiées. Elles ne doivent en aucun cas faire apparaître en clair les noms des personnes concernées, ni leur adresse, ni d'autre information permettant une identification directe.

Les patients inclus seront codifiés par les premières lettres du nom et du prénom du patient, accompagnées d'un numéro codé propre à la recherche indiquant l'ordre d'inclusion des patients.

Le numéro de code et le numéro de téléphone des patients inclus seront enregistrés sur une interface internet sécurisée permettant une programmation de l'appel dans les délais impartis par le protocole. Les informations fournies au prestataire du serveur vocal, à savoir les coordonnées téléphoniques et le numéro d'inclusion du patient, ne seront pas conservées par le prestataire et seront détruites au fur et à mesure des appels aboutis. La base de données de l'étude ne conservera que les réponses fournies par les patients lors de l'administration de la check-list.

Pour les patients qui auront signifié leur opposition à participer à l'étude en appelant le numéro de téléphone gratuit, aucun fichier électronique ne sera créé. Le refus sera noté dans le dossier source du patient sur le site où il doit se faire opérer.

11.1.2. Documents et données sources

Les données sources proviennent du dossier médical du patient inclus et du programme opératoire enregistré qu'il soit informatisé ou « papier ».

Les réponses des patients sur serveur vocal seront également considérés comme données sources, le fichier de ce données seront transmises à l'Unité de recherche clinique sous forme de fichier électronique.

11.2. CONTROLE ET ASSURANCE DE LA QUALITE

11.2.1. Consignes pour le recueil des données

Toutes les informations requises par le protocole doivent être consignées sur les cahiers d'observation. Ces données doivent être recueillies et enregistrées au fur et à mesure qu'elles sont obtenues, et transcrites dans ces cahiers de façon exacte, complète et lisible.

Les données de cette recherche seront collectées sur un cahier d'observation électronique.

11.2.2. Contrôle de qualité

L'investigateur met les documents et les données individuelles strictement nécessaires au suivi, au contrôle de qualité et à l'audit de cette recherche à la disposition des personnes chargées du contrôle de qualité et dûment mandatées par le gestionnaire à cet effet.
La(les) personne(s) mandatée par le gestionnaire visite(nt) de façon régulière chaque centre, lors de la mise en place de la recherche, une ou plusieurs fois en cours de recherche selon le rythme des inclusions et en fin de recherche. Lors de ces visites, les éléments suivants seront rappelés :

- protection et sécurité des personnes,
- respect du protocole de la recherche, des procédures qui y sont définies et des textes réglementaires en vigueur,
- qualité des données recueillies dans le cahier d'observation : exactitude, données manquantes,
- gestion des produits éventuels et des prélèvements.

Toute visite fera l'objet d'un rapport de visite par compte-rendu écrit.

11.2.3. Audit

Un audit peut être réalisé à tout moment par des personnes mandatées par le gestionnaire et indépendantes des responsables de la recherche. Il a pour objectif de s'assurer de la qualité de la recherche, de la validité de ses résultats et du respect de la loi et des règlementations en vigueur.

Les personnes qui dirigent et surveillent la recherche acceptent de se conformer aux exigences du gestionnaire et à l'autorité compétente en ce qui concerne un audit ou une inspection de la recherche.

L'audit pourra s'appliquer à tous les stades de la recherche, du développement du protocole à la publication des résultats et au classement des données utilisées ou produites dans le cadre de la recherche.

11.3. TRAITEMENT DES DONNEES ET CONSERVATION DES DOCUMENTS ET DES DONNEES RELATIVES A LA RECHERCHE

11.3.1. Traitement des données

La saisie des données est organisée sur un cahier d'observation électronique, et les personnels responsables de cette saisie sont les investigateurs ainsi que leur équipe, notamment les TECs dédiés à cette recherche. Les données seront d'accès restreint par un identifiant et un mot de passe, unique à chaque médecin investigateur en charge d'un patient. Les données saisies seront anonymisées et sécurisées, par cryptage lors du transfert de données. Les données seront vérifiées par un data manager. D'éventuelles queries seront éditées. Avant l'analyse statistique, la base de données sera gelée par le data manager. Cette base de données ayant donné lieu à l'analyse statistique fera l'objet d'un archivage par le responsable de l'analyse ou l'informaticien (support papier ou informatique). La durée de l'archivage est de 15 ans, sur serveur informatique avec sauvegarde quotidienne.

11.3.2. Conservation des documents et des données relatives à la recherche

Les documents d'une recherche entrant dans le cadre de la loi sur les recherches biomédicales doivent être archivés par toutes les parties pendant une durée de 15 ans après la fin de la recherche.(voir BPC, chapitre 8 : documents essentiels).

Cet archivage indexé comporte :

- Les copies de courrier de l'avis obligatoire du CPP
- Les versions successives du protocole (identifiées par le n° de version et la date de version),
- Les courriers de correspondance avec le promoteur,
- Les consentements signés des sujets sous pli cacheté (dans le cas de sujets mineurs signés par les titulaires de l'autorité parentale) avec la liste ou registre d'inclusion en correspondance,
- Le cahier d'observation complété et validé de chaque sujet inclus,
- Toutes les annexes spécifiques à l'étude,
- Le rapport final de l'étude provenant de l'analyse statistique et du contrôle qualité de l'étude (double transmis au promoteur).
- Les certificats d'audit éventuels réalisés au cours de la recherche

12. ASPECTS STATISTIQUES

L'analyse statistique sera assurée par le Centre d'Epidémiologie Clinique du Pr Ravaud (Hôpital Hôtel Dieu) sur les fichiers de données gelées, à l'aide du logiciel statistique SAS®.
Un plan d'analyse statistique sera rédigé et validé avant la revue en aveugle des données. Il sera proposé par le Centre d'Epidémiologie Clinique et revu par le Gestionnaire et l'Investigateur.
Les analyses à réaliser pourront être complétées lors de cette revue. La version finale du Plan d'Analyses Statistiques sera réalisée avant la levée d'aveugle. L'ensemble des versions sera conservé dans le dossier de l'étude.
Le Plan d'Analyses Statistiques pourra être révisé en cours d'étude, afin de prendre en compte les éventuelles modifications apportées au Protocole ou toute autre modification du déroulement de l'étude ayant un impact sur les analyses statistiques initialement prévues.
.
Pour chaque groupe, les critères qualitatifs seront décrits par leur effectif, pourcentage et données manquantes par modalité de réponse et les critères quantitatifs par leur effectif, moyenne, écart-type. En cas de critères quantitatifs à comportement asymétrique, ils seront présentés avec leur médiane et l'intervalle interquartile (25ème percentile- 75ème percentile).

12.1. JUSTIFICATION DE LA TAILLE DE L'ECHANTILLON

Sur la base des informations fournis par les centres participants sur 1 mois, le taux d'annulation dans le groupe sans intervention est estimé à environ 10%. L'objectif est de réduire le pourcentage d'annulation de 4% en valeur absolue avec l'intervention (soit un pourcentage d'annulation de 6% dans le groupe intervention). Pour un design classique avec 2 bras parallèles et un risque de première espèce fixé à 5%, il est nécessaire d'inclure environ 1193 patients par bras soit 2386 afin mettre en évidence une différence avec une puissance égale à 95%. Cependant, dans cette étude, d'autres éléments sont à prendre en considération pour le calcul du nombre de sujets : corrélation intra centre mais aussi la variabilité inter centre, le nombre de centres, la taille des centres. En considérant un facteur d'inflation important (1.5), il est donc nécessaire d'inclure au minimum 3580 patients au total.
Nous souhaitons augmenter le nombre de patients à inclure dans notre essai pour 2 raisons

1) Nous avons des patients inclus à tort qu'il faudrait remplacer (estimés à 150 patients).
2) Nous avons environ 10% de données manquantes et nous voudrions en tenir compte et augmenter la taille de notre échantillon.

Par conséquent il faudrait prévoir d'inclure 4090 patients au lieu de 3580 soit 510 patients supplémentaires.

12.2. DESCRIPTION DES METHODES STATISTIQUES PREVUES Y COMPRIS DU CALENDRIER DES ANALYSES INTERMEDIAIRES PREVUES

L'analyse réalisée sera en intention de traiter c'est-à-dire, tous les patients randomisés seront analysés dans leur bras de randomisation quelle que soit l'intervention reçue. L'analyse du critère de jugement principal sera effectuée de la manière suivante. Un modèle linéaire généralisé mixte (avec un lien logit) sera utilisé afin de comparer les pourcentages d'annulation entre les 2 interventions. L'effet aléatoire sera le centre alors l'intervention et les autres variables d'ajustement seront des effets fixes. La matrice de variance-covariance entre les effets aléatoires sera à symétrie de composition (la corrélation est identique entre chaque paire de patients d'un même cluster). Des interactions pourront être introduites dans le modèle et plus particulièrement l'interaction entre le centre et le temps, celle entre le centre et l'intervention (l'effet de l'intervention est-il différent selon les centres), celle entre l'intervention et le temps (l'effet de l'intervention avec le temps).

L'analyse principale sera répétée sur les critères secondaires. Les tests effectués seront considérés comme significatifs si degrés de signification inférieurs à 5%.

13. ASPECTS ETHIQUES ET LEGAUX

13.1. DECLARATION INDIQUANT QUE LA RECHERCHE SERA CONDUITE CONFORMEMENT AU PROTOCOLE, AUX DISPOSITIONS LEGISLATIVES ET REGLEMENTAIRES EN VIGUEUR

Le gestionnaire et la(les) personne(s) qui dirige(nt) et surveille(nt) la recherche s'engagent à ce que cette recherche soit réalisée en conformité avec la loi n°2004-806 du 9 août 2004 relative à la politique de santé publique et les dispositions réglementaires en vigueur. (Articles L1121-1, 2° alinéa et R1121-3 du Code de la santé publique).

Les données enregistrées à l'occasion de cette recherche feront l'objet d'un traitement informatisé dans le respect de la loi n°78-17 du 6 janvier 1978 relative à l'informatique, aux fichiers et aux libertés modifiée.

La recherche sera conduite conformément au présent protocole.

13.2. EVALUATION ETHIQUE DES MODALITES PARTICULIERES DE SURVEILLANCE PREVUES PAR LE PROTOCOLE

Les modalités particulières de surveillance ajoutées par la recherche ont fait l'objet d'une évaluation éthique par le CPP IDF I, Hôtel Dieu. Elles ne comportent que des risques et contraintes négligeables pour les personnes qui se prêtent à cette recherche.

Toutefois, si des événements indésirables liés aux modalités particulières de surveillance survenaient au cours de la recherche, le gestionnaire a prévu un circuit de notification et de gestion de ces événements (Cf. section **10.1**)

13.3. OBLIGATIONS LEGALES (ROLE DU GESTIONNAIRE, CPP, CCTIRS, CNIL)

13.3.1. Rôle du gestionnaire

L'Assistance Publique des Hôpitaux de Paris (AP-HP) est le gestionnaire de cette recherche. Le DRCD en est le représentant.

La personne physique ou morale qui prend l'initiative de cette recherche, en assure la gestion et vérifie que son financement est prévu, est dénommée gestionnaire.

Il soumet le dossier à l'avis du comité de protection des personnes concerné.

Conformément à la loi n°78-17 du 6 janvier 1978 modifiée, il adresse une demande d'avis au Comité Consultatif sur le Traitement de l'Information en matière de Recherche dans le domaine de la Santé (CCTIRS) et une demande d'autorisation à la Commission Nationale de l'Informatique et des Libertés (CNIL) pour le traitement des données enregistrées à l'occasion de cette recherche.

13.3.2. Soumission au CPP

Cette recherche a obtenu l'avis favorable du Comité de Protection des Personnes Ile de France I, Hôtel Dieu le 30/01/2012.

L'avis du comité mentionné ci-dessus est notifié dans la note d'information destinée aux personnes concernées.

13.3.3. Avis du CCTIRS et autorisation à la CNIL

Cette recherche est soumise à la loi n°78-17 du 6 janvier 1978 relative à l'informatique, aux fichiers et aux libertés modifiée.
Par conséquent, le traitement des données collectées dans le cadre des recherches multicentriques est subordonné à la saisine du Comité consultatif sur le traitement de l'information en matière de recherche dans le domaine de la santé (CCTIRS) pour avis puis à l'autorisation de la Commission Nationale de l'Informatique et des Libertés (CNIL).

Les informations relatives aux droits des personnes participant à cette recherche (droit d'accès et de rectification, droit d'opposition à la transmission des données couvertes par le secret professionnel susceptibles d'être utilisées dans le cadre de cette recherche) sont intégrées dans la note d'information destinée au patient.

13.3.4. Modification substantielle au protocole

L'investigateur coordonnateur informe le DRCD de tout projet de modification du protocole. Toute modification substantielle sera soumise par le gestionnaire de cette recherche au CPP pour avis.

13.3.5. Rapport final de la recherche ou publication

Le rapport final de la recherche ou la publication sera soumis à chacun des centres participants pour avis. La version finale sera adressée dans les meilleurs délais au gestionnaire après la fin effective de la recherche.

13.3.6. Propriété des données

L'AP-HP est propriétaire des données et aucune utilisation ou transmission à un tiers ne peut être effectuée sans son accord préalable.

13.3.7. Règles relatives à la publication

L'AP-HP sera mentionné dans les affiliations du ou des auteurs des publications qui résulteront de cette recherche ainsi que le gestionnaire AP-HP (DRCD) et la source de financement puisqu'il s'agit d'un appel d'offre PREQHOS.
Les modalités d'affiliation et de mention du promoteur et du financeur seront définis comme telles :

1) Mention de l'affiliation de l'AP-HP :
L'institution AP-HP apparaîtra sous le sigle « AP-HP » en premier dans l'adresse suivi précisément par : AP-HP, hôpital, service, ville, code postal, France ;

2) Mention du gestionnaire AP-HP (DRCD) dans les ”acknowledgments” du manuscrit
”The sponsor was Assistance Publique – Hôpitaux de Paris (Département de la Recherche Clinique et du Développement)”

3) Mention du financeur dans les ”acknowledgments” du manuscrit
“The study was funded by a grant from Programme de Recherche en Qualité Hospitalière - PREQHOS 2011 (Ministère de la Santé)”

Cette recherche sera enregistrée sur le site http://clinicaltrials.gov/ sous le n° *NCT01732159*.

14. BIBLIOGRAPHIE

1. Recommandations formalisées d'experts. Prise en charge anesthésiques des patients en hospitalisation ambulatoire. SFAR 2009. <http://www.sfar.org/categorie/10/conf-rences-experts-rfe/1>.
2. Rapport de la Mission National d'Expertise et d'Audits Hospitaliers (MEAH) 2007. La Chirurgie Ambulatoire. <http://www.meah.sante.gouv.fr/meah/index.php?id=30>.
3. Tung A, Dexter F, Jakubczyk S, Glick DB. The limited value of sequencing cases based on their probability of cancellation. *Anesth Analg.* Sep 2010;111(3):749-756.
4. Pollard JB, Olson L. Early outpatient preoperative anesthesia assessment: does it help to reduce operating room cancellations? *Anesth Analg.* Aug 1999;89(2):502-505.
5. McWhinnie DL, Michaels JA, Collin J, Morris PJ. Resource implications of cancelled operations. *BMJ.* Jan 8 1994;308(6921):138-139.
6. Argo JL, Vick CC, Graham LA, Itani KM, Bishop MJ, Hawn MT. Elective surgical case cancellation in the Veterans Health Administration system: identifying areas for improvement. *Am J Surg.* Nov 2009;198(5):600-606.
7. Weiser TG, Haynes AB, Dziekan G, Berry WR, Lipsitz SR, Gawande AA. Effect of a 19-item surgical safety checklist during urgent operations in a global patient population. *Ann Surg.* May 2010;251(5):976-980.
8. Khuri SF, Henderson WG, Daley J, et al. Successful implementation of the Department of Veterans Affairs' National Surgical Quality Improvement Program in the private sector: the Patient Safety in Surgery study. *Ann Surg.* Aug 2008;248(2):329-336.
9. Semel ME, Resch S, Haynes AB, et al. Adopting a surgical safety checklist could save money and improve the quality of care in U.S. hospitals. *Health Aff (Millwood).* Sep 2010;29(9):1593-1599.
10. Sewell M, Adebibe M, Jayakumar P, et al. Use of the WHO surgical safety checklist in trauma and orthopaedic patients. *Int Orthop.* Aug 21 2010.
11. Lingard L, Regehr G, Orser B, et al. Evaluation of a preoperative checklist and team briefing among surgeons, nurses, and anesthesiologists to reduce failures in communication. *Arch Surg.* Jan 2008;143(1):12-17; discussion 18.
12. Haynes AB, Weiser TG, Berry WR, et al. A surgical safety checklist to reduce morbidity and mortality in a global population. *N Engl J Med.* Jan 29 2009;360(5):491-499.
13. Levin DC. A surgical safety checklist. *N Engl J Med.* May 28 2009;360(22):2374; author reply 2374-2375.
14. Martin IC, Mason M, Findlay G. A surgical safety checklist. *N Engl J Med.* May 28 2009;360(22):2372-2373; author reply 2374-2375.
15. McCambridge J, Kypri K, Elbourne DR. A surgical safety checklist. *N Engl J Med.* May 28 2009;360(22):2373-2374; author reply 2374-2375.
16. Sanders RD, Jameson SS. A surgical safety checklist. *N Engl J Med.* May 28 2009;360(22):2373; author reply 2374-2375.
17. Schulz KF, Altman DG, Moher D. CONSORT 2010 statement: updated guidelines for reporting parallel group randomized trials. *Ann Intern Med.* Jun 1;152(11):726-732.
18. Schulz KF, Altman DG, Moher D. CONSORT 2010 Statement: updated guidelines for reporting parallel group randomised trials. *BMC Med.*8:18.
19. Schulz KF, Altman DG, Moher D. CONSORT 2010 Statement: updated guidelines for reporting parallel group randomised trials. *Trials.*11:32.

20. Moher D, Hopewell S, Schulz KF, et al. CONSORT 2010 explanation and elaboration: updated guidelines for reporting parallel group randomised trials. *BMJ*.340:c869.

21. Schulz KF, Altman DG, Moher D. CONSORT 2010 statement: updated guidelines for reporting parallel group randomised trials. *BMJ*.340:c332.

22. Wood L, Egger M, Gluud LL, et al. Empirical evidence of bias in treatment effect estimates in controlled trials with different interventions and outcomes: meta-epidemiological study. *Bmj*. Mar 15 2008;336(7644):601-605.

15. ANNEXES

ANNEXE 1
LISTE DES INVESTIGATEURS PRINCIPAUX

Centres		Liste des personnes (Veuillez indiquer le nom et prenom)	
N°	Nom et Adresse complete		
**1**	Hôpitaux Universitaires Paris centre<br>Site Port-Royal<br>Avenue de l'observatoire<br>75014 Paris	**Pr Jean-Pierre Bethoux**<br><br>**Dr Sonia Gaucher**	Service de Chirurgie Générale, Plastique et Ambulatoire<br><br>Mail : jp.bethoux@htd.aphp.fr<br>sonia.gaucher@htd.aphp.fr<br>Tel : 01 58 41 37 82
**2**	Hôpital Saint Antoine<br>184, rue du Faubourg Saint-Antoine<br>75571 PARIS Cedex 12	**Pr Marc BEAUSSIER**	Service d'Anesthésie et Chirurgie Ambulatoire<br><br>marc.beaussier@sat.aphp.fr
**3**	Hôpital Jean Verdier<br>Avenue du 14 Juillet<br>93140 Bondy	**Pr Corinne VONS**	Service de Chirurgie et d'Anesthésie Ambulatoire<br><br>corinne.vons@jvr.aphp.fr
**4**	Hôpital Tenon<br>4, rue de la Chine –<br>75970 PARIS Cedex 20	**Dr Michel MAILLET**	Unité d'Anesthésie et de Chirurgie Ambulatoire<br><br>michel.maillet@tnn.aphp.fr
**5**	Hôpital Européen Georges Pompidou<br>20, rue Leblanc<br>75908 PARIS Cedex 15	**Pr Laurent LANTIERI**	Unité d'Anesthésie et de Chirurgie Ambulatoire<br><br>laurent.lantieri@egp.aphp.fr

**6**	Hôpital Ambroise Paré<br>9, avenue Charles De Gaulle – 92100 Boulogne-Billancourt.	**Pr. Philippe HARDY**<br>**Dr. Guy KUHLMAN**	Hôpital de jour medico-chirurgical<br>philippe.hardy@apr.aphp.fr<br>guy.kuhlman@apr.aphp.fr
**7**	Hôpital Trousseau<br>26, avenue du Dr. Arnold-Netter – 75571 Paris Cedex 12.	**Dr. Frédéric AUBER**	Unité de Chirurgie et d'Anesthésie Ambulatoire<br>frederic.auber@trs.aphp.fr
**8**	Hôpital Bicêtre<br>78, rue du Général Leclerc – 94275 Le Kremlin-Bicêtre Cedex	**Dr Véronique MOLINA**	Unité de chirurgie ambulatoire<br>veronique.molina@bct.aphp.fr
**9**	Hôpital Robert Debré<br>48, boulevard Sérurier – 75935 Paris Cedex 19.	**Pr. Yves NIVOCHE**	Hôpital de Jour Chirurgie<br>yves.nivoche@rdb.aphp.fr
**10**	Hôpital Bichat<br>46, rue Henri-Huchard – 75877 Paris Cedex 18	**Dr. Frédérique SERVIN**	Hôpital de jour de chirurgie ambulatoire<br>frederique.servin@bch.aphp.fr
**11**	Hôpital Avicenne<br>125, rue de Stalingrad – 93009 Bobigny Cedex.	**Pr. Christophe BAILLARD**<br>**Pr Richard DOUARD**<br>**Pr Philippe WIND**	Unité de chirurgie ambulatoire<br>christophe.baillard@avc.aphp.fr<br>richard.douard@avc.aphp.fr<br>philippe.wind@avc.aphp.fr

ANNEXE 2
CHECK-LIST

Bonjour,
Vous, ou votre enfant, allez vous faire opérer en chirurgie ambulatoire à l'hôpital ….. Afin d'organiser au mieux l'opération, merci de répondre aux questions suivantes.

Vous pouvez choisir d'écouter les questions dans les langues suivantes : français, anglais, mandarin, arabe ou portugais.

Ce serveur vocal n'enregistre aucun commentaire en dehors des réponses attendues.

Si vous ne voulez pas répondre à ce système vocal, merci de taper 1 sur votre clavier de téléphone et une personne vous contactera.

1. ***Vous allez être opéré en chirurgie ambulatoire dans les jours qui viennent. Est ce que vous maintenez la date prévue de votre opération?*** *(Votre enfant va être opéré en chirurgie ambulatoire dans les jours qui viennent. Est ce que vous maintenez la date prévue de son opération?)*
    a. Si oui, le TEC/système vocal passe à la question suivante.
    b. Si non, le TEC/ système vocal alerte l'UCA et le patient est déprogrammé.

Mesure correctrice si réponse 1b: Le service renseigne la raison de l'annulation en précisant s'il s'agit d'une annulation définitive ou d'un report. Le service organise la suite de la prise en charge du patient au décours de l'annulation (nouvelle date opératoire ou nouveau rendez vous en consultation avec le chirurgien référent, …).

2. ***Avez-vous un accompagnant majeur pour le retour à domicile?*** *(Avez-vous un moyen de retour à domicile organisé avec deux adultes (les 2 parents ou un parent et un autre adulte accompagnant) et la présence d'au moins un adulte la première nuit post-opératoire?)*
    a. Si oui, le TEC /système vocal passe à la question suivante.
    b. Si non, le TEC/ système vocal annonce au patient qu'il ne peut pas être opéré s'il n'a pas d'accompagnant, qu'il risque d'être déprogrammé s'il n'en trouve pas et qu'il doit prévenir l'UCA le plus rapidement possible (avant J-1). Puis le TEC/système vocal passe à la question suivante.

3. ***Avez-vous fait vos formalités administratives pour votre admission?*** *(Avez-vous fait les formalités administratives pour l'admission de votre enfant?)*
    a. Si oui, le TEC/système vocal passe à la question suivante.
    b. Si non, le TEC/ système vocal annonce au patient que si l'admission n'est pas réalisée avant l'opération, celle-ci sera retardée, voire annulée, et rappelle que pour réaliser l'admission, le patient doit se munir de la liste de papiers suivants: CNI, carte vitale/attestation CMU, attestation sécurité sociale, attestation mutuelle, autorisation d'opérer si mineur ou majeur protégé. Le TEC/système vocal ajoute que si le patient ne peut faire son admission en raison d'un problème de prise en charge, le patient doit contacter l'UCA le

plus rapidement possible. Puis le TEC/système vocal passe à la question suivante.

4. ***Est ce que votre état de santé a changé depuis la dernière consultation à l'hôpital, avec un éventuel changement de votre traitement habituel?*** *(Est ce que l'état de santé de votre enfant a changé depuis la dernière consultation à l'hôpital, avec un éventuel changement de votre traitement habituel ?)*
   a. Si non, le TEC/système vocal passe à la question suivante.
   b. Si oui, le TEC/ système vocal annonce au patient qu'il doit appeler l'UCA le plus rapidement possible pour vérifier que la maladie et le nouveau traitement n'interfèrent pas avec l'intervention et que cette dernière est toujours réalisable. Puis le TEC/système vocal passe à la question suivante.

Mesure correctrice si réponse 4bii: Le service renseigne la nature du problème de santé et du nouveau traitement, puis contacte le médecin référent (anesthésiste ou chirurgien selon la nature du problème de santé et du nouveau traitement), puis recontacte le patient avec l'avis médical.

5. ***Est-ce que le médecin ou le chirurgien vous a demandé de modifier votre traitement habituel en vue de votre opération?*** *(Est-ce que le médecin ou le chirurgien vous a demandé de modifier le traitement habituel de votre enfant en vue de son opération?)*
   a. Si non, le TEC/système vocal passe à la question suivante.
   b. Si oui, le TEC/système vocal pose la question *Avez-vous des questions sur les modifications de votre traitement? (Avez-vous des questions sur les modifications du traitement de votre enfant?)*
      i. Si non, le TEC passe à la question suivante.
      ii. Si oui, le TEC/ système vocal demande au patient d'appeler l'UCA le plus rapidement possible. Puis le TEC/système vocal passe à la question suivante.

Mesure correctrice si réponse 5bii: Le service renseigne le traitement concerné, puis contacte le médecin référent (anesthésiste ou chirurgien selon la nature du traitement concerné), puis recontacte le patient avec l'avis médical.

6. ***Est-ce que le médecin ou le chirurgien vous a demandé de réaliser des examens en vue de votre opération ?****(Est-ce que le médecin ou le chirurgien a demandé de faire réaliser des examens à votre enfant en vue de son opération?)*
   a. Si non, le TEC/système vocal passe à la question suivante.
   b. Si oui, le TEC/système vocal pose la question suivante : *Avez vous fait les examens demandés?(Avez vous fait les examens demandés à votre enfants?)*
      i. Si non, le TEC/ système vocal rappelle au patient que si des examens ont été prescrits, c'est parce qu'ils sont nécessaires pour l'opération; que si les examens ne sont pas réalisé, l'opération risque d'être annulée; que si le patient est dans l'impossibilité de les réaliser, il doit contacter l'UCA le plus rapidement possible. Puis le TEC/système vocal passe à la question suivante.

ii. Si oui, le TEC/système vocal dit *Pensez à rapporter tous les examens le jour de l'intervention* puis le TEC/système vocal passe à la question suivante.

7. ***Avez-vous des questions sur les consignes pour rester à jeun?*** *(Avez vous des questions sur les consignes pour que votre enfant reste à jeun?)*
   - *a.* Si non, le TEC/système vocal clôture le questionnaire.
   - b. Si oui, le TEC/système vocal demande au patient de rappeler l'UCA pour avoir des informations sur les consignes. Puis le TEC/système vocal clôture le questionnaire.

Mesure correctrice si réponse 7b: Le service rappelle au patient les consignes de jeûne, et donne, si besoin, des précisions en cas de régime particulier à respecter (régime sans résidu par exemple).
