# Supplementary material for: Assessment of a Standardized Pre-Operative Telephone Checklist Designed to Avoid Late Cancellation of Ambulatory Surgery: The AMBUPROG Multicenter Randomized Controlled Trial
Source: PLoS One. 2016 Feb 1;11(2):e0147194. doi: 10.1371/journal.pone.0147194 (PMC4734771; doi:10.1371/journal.pone.0147194)
Supplement: S2 Protocol — (PDF) [file pone.0147194.s003.pdf]

AMBUPROG synopsis

**Title of clinical trial**	Impact of a checklist on the rate of late cancellation in ambulatory surgery
**Acronym**	AMBUPROG
**Protocol version**	Version 4.0 of June 20, 2013
**Funding**	French Health Ministry, winner of the tender PREQHOS 2011
**Administrator**	Assistance Publique-Hôpitaux de Paris, Paris, France
**Supervisor**	Pr Jean-Pierre Béthoux
**Purpose of clinical trial**	Assessment of a standardized pre-operative telephone checklist on the rate of late cancellation in ambulatory surgery
**Population study**	All patients scheduled for ambulatory surgery without any exclusion criteria
**Expected number of centers**	11 university hospital ambulatory surgery units of Assistance Publique-Hôpitaux de Paris, Paris, France
**Expected sample size**	4 090 patients<br>- Group control = 2 045 patients<br>- Group intervention = 2 045 patients
**Primary objective**	Assessment of a standardized pre-operative telephone checklist on the rate of late cancellation in ambulatory surgery the day before the surgery, or the day of the surgery
**Inclusion criteria**	1. Patient (adult or minor) scheduled for ambulatory surgery in multidisciplinary ambulatory surgery unit<br>2. Surgery under general anesthesia, locoregional anesthesia and/or sedation<br>3. Could be reached by telephone<br>4. Not opposed to participation
**Exclusion criteria**	1. Patient scheduled for non-surgical gastrointestinal endoscopy<br>2. Patient scheduled for termination of pregnancy<br>3. Emergency ambulatory surgery<br>4. Surgery under local anesthesia
**Endpoints**	Primary endpoints:<br>- The percentage of late cancellation, defined as cancellation the day before surgery or on the day of surgery<br>Secondary endpoints:<br>- The percentage of cancellations the day before the surgery<br>- The percentage of cancellations on the day of surgery<br>- The percentage of conversion to conventional hospitalization
**Trial design**	Multicenter, two-arm, parallel-group, open-label randomized controlled trial
**Duration of the study**	Duration of the period inclusion period : 33 months<br>Duration of the patient participation : less than or equal to 1 month<br>Total duration of the study : 3 years
