# Supplementary material for: Assessment of a Standardized Pre-Operative Telephone Checklist Designed to Avoid Late Cancellation of Ambulatory Surgery: The AMBUPROG Multicenter Randomized Controlled Trial
Source: PLoS One. 2016 Feb 1;11(2):e0147194. doi: 10.1371/journal.pone.0147194 (PMC4734771; doi:10.1371/journal.pone.0147194)
Supplement: S3 Protocol — (PDF) [file pone.0147194.s004.pdf]

ASSISTANCE PUBLIQUE HÔPITAUX DE PARIS

AMBUPROG

Impact d'une « check-list » sur le nombre de déprogrammations tardives des patients en chirurgie ambulatoire

Protocole de recherche visant à évaluer les soins courants

ADDENDUM 1, version 1.0 du 31/07/2013 (MS4)

Au protocole version N°4-0 du 20/06/2013

Codes projet : **PHRQ1145 / K110601 / N°ID RCB : 2011-A01647-34**

Investigateur coordonnateur :

Pr Jean-Pierre BETHOUX
Service de Chirurgie Générale, Plastique et Ambulatoire
Hôpitaux Universitaires Paris Centre
Nouveau Bâtiment Port-Royal
53 Avenue de l'Observatoire
75014 Paris

☎ : 01 58 41 37 82-

E-mail : jean-pierre.bethoux@cch.aphp.fr

Gestionnaire :

AP-HP- DRCD
1, avenue Claude Vellefaux
75010 PARIS
Référent projet : Ludovic DYEN
☎ : 01.44.84.17.43 - 🖷 : 01.44.84.17.01

E-mail : ludovic.dyen@sls.aphp.fr

URC Cochin-Necker
Référent projet : Sabine HELFEN
GH Cochin – site Tarnier
89 rue d'Assas – 75006 Paris
☎ : 01.58.41.11.90 - 🖷 : 01.58.41.11.83

E-mail : sabine.helfen@cch.aphp.fr

Département de la Recherche Clinique et du Développement
DIRC Ile de France
Hôpital Saint Louis 75010 PARIS

Page de SIGNATURE D'UN PROTOCOLE de recherche visant à évaluer les soins courants

Codes de la Recherche : PHRQ1145 / K110601 / N° ID RCB : 2011-A01647-34

Titre : AMBUPROG : Impact d'une « check-list » sur le nombre de déprogrammations tardives des patients en chirurgie ambulatoire

ADDENDUM 1 version 1.0 du 31/07/2013 (MS4) au protocole version N° 4-0 du 20/06/2013

La recherche sera conduite conformément au protocole et aux dispositions législatives et réglementaires en vigueur.

L'investigateur coordonnateur :
Pr Jean-Pierre BETHOUX
Service de Chirurgie Générale, Plastique et Ambulatoire
Hôpitaux universitaires Paris Centre
Nouveau Bâtiment Port-Royal
53 Avenue de l'Observatoire
75014 Paris

Date : 10/09/2013
Signature :

Le responsable scientifique :
Dr Isabelle BOUTRON
Centre d'épidémiologie Clinique INSERM U738
Hôpital Hôtel Dieu
1, place du Parvis Notre-Dame
75181 Paris cedex 4

Date : 26/9/13
Signature :

Le gestionnaire :
Christophe MISSE
Directeur du DRCD
Assistance Publique – Hôpitaux de Paris
Département de la Recherche Clinique et du Développement
Hôpital Saint Louis
75010 PARIS

Date : 16 OCT. 2013
Signature :

La recherche a reçu :

- un avis favorable initial du CPP IDF I, Hôtel Dieu en date du 30/01/2012,
- l'avis favorable pour la MS 1, 23/07/2012,
- l'avis favorable pour la MS 2, le 12/02/2013,
- l'avis favorable pour la MS 3, le 27/06/2013 et,
- l'avis favorable pour la MS 4, le 04/09/2013.

TABLE DES MATIÈRES

1. RESUME SYNOPTIQUE **4**

2. JUSTIFICATION ADDENDUM **5**

3. ANNEXES **6**

ANNEXE 1 .... **6**

1. RESUME SYNOPTIQUE

**Titre**	**AMBUPROG**<br>Impact d'une « check-list » sur le nombre de déprogrammations tardives des patients en chirurgie ambulatoire.
**Version du protocole**	**version 4.0 du 20/06/2013**
**Source de financement**	Lauréat de l'appel d'offre PREQHOS
**Gestionnaire**	Assistance Publique – Hôpitaux de Paris (AP-HP)
**Investigateur Coordonnateur**	Pr Jean-Pierre BETHOUX
**Nature du soin courant évalué**	Impact d'une check-list administrée aux patients sur les déprogrammations tardives en chirurgie ambulatoire
**Population concernée**	Tous les patients majeurs et mineurs programmés pour une chirurgie ambulatoire sauf critères d'exclusion
**Nombre de centres prévus**	11 centres hospitaliers de l'AP-HP
**Nombre de patients prévus**	4090 avec<br>- Groupe contrôle : 2045<br>- Groupe avec « check-list » : 2045
**Objectif principal**	Evaluation de l'impact d'une « check-list » sur le nombre de déprogrammations tardives (la veille ou le jour de l'intervention) des patients en chirurgie ambulatoire
**Critères d'inclusion**	1. Patient (adulte ou mineur) pour lequel une intervention chirurgicale ambulatoire est programmée dans une UCA polyvalente<br>2. Chirurgie sous anesthésie générale ou loco-régionale ou neuroleptanalgésie<br>3. Patient joignable par téléphone<br>4. Patient ne s'opposant pas à sa participation dans l'étude
**Critères de non inclusion**	1. Patient programmé en UCA pour une endoscopie non chirurgicale<br>2. Chirurgie ambulatoire programmée pour une orthogénie<br>3. Chirurgie ambulatoire programmée en urgence<br>4. Chirurgie ambulatoire réalisée sous anesthésie locale
**Critères d'évaluation**	- Le nombre de déprogrammation des patients en chirurgie ambulatoire la veille ou le jour de l'admission (J-1 ou J0)<br>- Le nombre de déprogrammation des patients en chirurgie ambulatoire à J-1<br>- Le nombre de déprogrammation des patients en chirurgie ambulatoire à J0<br>- Le nombre de passage en hospitalisation conventionnelle
**Méthodologie de la recherche**	Etude nationale de soins courants, multicentrique, randomisée en ouvert
**Durée de la recherche**	- Durée de la période d'inclusion : 33 mois<br>- Durée de participation du patient : inférieure ou égal à 1 mois<br>- Durée totale de la recherche : 3 ans

2. JUSTIFICATION ADDENDUM

Cet addendum 1 v1.0 du 31/07/2013 au protocole v4.0 du 20/06/2013 s'inscrit dans le cadre de la modification substantielle N° 4 et porte sur le changement d'investigateur principal du centre 7/Hôpital Trousseau-Paris : Dr Auber a quitté le service fin juillet 2013. Il est remplacé par le Dr Delaporte-Cerceau qui était déjà impliquée sur l'étude comme co-investigatrice.

3. ANNEXES

ANNEXE 1. LISTE DES INVESTIGATEURS PRINCIPAUX

Centres		Liste des personnes (Veuillez indiquer le nom et prenom)	
N°	Nom et Adresse complete		
1	**Hôpitaux Universitaires Paris centre**<br>Site Port-Royal<br>Avenue de l'observatoire<br>75014 Paris	**Pr Jean-Pierre Bethoux**<br><br>**Dr Sonia Gaucher**	Service de Chirurgie Générale, Plastique et Ambulatoire<br>jp.bethoux@htd.aphp.fr<br>sonia.gaucher@htd.aphp.fr<br>Tel : 01 58 41 37 82
2	**Hôpital Saint Antoine**<br>184, rue du Faubourg Saint-Antoine<br>75571 PARIS Cedex 12	**Pr Marc BEAUSSIER**	Service d'Anesthésie et Chirurgie Ambulatoire<br>marc.beaussier@sat.aphp.fr
3	**Hôpital Jean Verdier**<br>Avenue du 14 Juillet<br>93140 Bondy	**Pr Corinne VONS**	Service de Chirurgie et d'Anesthésie Ambulatoire<br>corinne.vons@jvr.aphp.fr
4	**Hôpital Tenon**<br>4, rue de la Chine –<br>75970 PARIS Cedex 20	**Dr Michel MAILLET**	Unité d'Anesthésie et de Chirurgie Ambulatoire<br>michel.maillet@tnn.aphp.fr
5	**Hôpital Européen Georges Pompidou**<br>20, rue Leblanc<br>75908 PARIS Cedex 15	**Pr Laurent LANTIERI**	Unité d'Anesthésie et de Chirurgie Ambulatoire<br>laurent.lantieri@egp.aphp.fr
6	**Hôpital Ambroise Paré**<br>9, avenue Charles De Gaulle –<br>92100 Boulogne-Billancourt.	**Pr. Philippe HARDY**<br>**Dr. Guy KUHLMAN**	Hôpital de jour medico-chirurgical<br>philippe.hardy@apr.aphp.fr<br>guy.kuhlman@apr.aphp.fr
7	**Hôpital Trousseau**<br>26, avenue du Dr. Arnold-Netter –<br>75571 Paris Cedex 12.	**Dr Sonia Delaporte-Cerceau**	Unité de Chirurgie et d'Anesthésie Ambulatoire<br><br>sonia.delaporte-cerceau@trs.aphp.fr
8	**Hôpital Bicêtre**<br>78, rue du Général Leclerc –<br>94275 Le Kremlin-Bicêtre Cedex	**Dr Véronique MOLINA**	Unité de chirurgie ambulatoire<br>veronique.molina@bct.aphp.fr
9	**Hôpital Robert Debré**<br>48, boulevard Sérurier – 75935 Paris Cedex 19.	**Pr. Yves NIVOCHE**	Hôpital de Jour Chirurgie<br>yves.nivoche@rdb.aphp.fr
10	**Hôpital Bichat**<br>46, rue Henri-Huchard – 75877 Paris Cedex 18	**Dr. Frédérique SERVIN**	Hôpital de jour de chirurgie ambulatoire<br>frederique.servin@bch.aphp.fr
11	**Hôpital Avicenne**<br>125, rue de Stalingrad – 93009 Bobigny Cedex.	**Pr. Christophe BAILLARD**<br>**Pr Richard DOUARD**<br>**Pr Philippe WIND**	Unité de chirurgie ambulatoire<br>christophe.baillard@avc.aphp.fr<br>richard.douard@avc.aphp.fr<br>philippe.wind@avc.aphp.fr
