# Supplementary material for: Assessment of a Standardized Pre-Operative Telephone Checklist Designed to Avoid Late Cancellation of Ambulatory Surgery: The AMBUPROG Multicenter Randomized Controlled Trial
Source: PLoS One. 2016 Feb 1;11(2):e0147194. doi: 10.1371/journal.pone.0147194 (PMC4734771; doi:10.1371/journal.pone.0147194)
Supplement: S4 Protocol — (PDF) [file pone.0147194.s005.pdf]

COMITE DE PROTECTION DES PERSONNES - Ile de France 1

CPP ILE DE France I – N°IRB : 00008522 – responsable administrative : Hélène de Crécy
Hôtel-Dieu – 1, Place du Parvis Notre-Dame – 75181 PARIS cedex 04
Tél. : 01 42 34 80 52 – Port. 06 63 34 80 52 – Fax : 01 42 34 86 11 – E-Mail : cppiledefrance1@orange.fr – E-Mail : ccp.prb@htd.aphp.fr

Ludovic DYEN - Chef de Projet
DIRC Ile de France
Assistance Publique-Hôpitaux de Paris
(Direction de la Recherche Clinique et du Développement)
Carré Historique,
Hôpital Saint Louis, Secteur Gris, Porte 23
1 Av. Claude Vellefaux
75475 Paris Cedex 10

Tel: +33 (0)1.44.84.17.43
Fax: +33 (0)1.44 84 17.01
Email: ludovic.dyen@sls.aphp.fr

Paris, le 31 janvier 2012

Nos références CPP Ile de France 1 - NUMERO DOSSIER : 2012-Janv.-12806

Le 3 janvier 2012, le comité a été saisi d'une demande initiale concernant le projet de recherche en soins courants intitulé : **AMBUPROG. Impact d'une "check-list" informatisée sur le taux de déprogrammation tardive des patients en chirurgie ambulatoire. Réf. Promoteur : PHRQ1145 – ID RCB 2011-A01647-34**

- **Promoteur** : Assistance Publique – Hôpitaux de Paris
- **Investigateur principal** : Investigateur Principal : Pr Jean-Pierre BETHOUX, Service de Chirurgie Générale Viscérale et Thoracique - Hôpital HOTEL DIEU, 1 PL DU PARVIS NOTRE-DAME, 75004 PARIS

Le Bureau a adopté ce jour, lundi 30 janvier 2012, la délibération suivante :

AVIS FAVORABLE

ONT PARTICIPE A LA DELIBERATION :

- PREMIER COLLEGE

- **Médecin ou personne qualifiée en matière de recherche biomédicale** : Elisabeth FRIJA ; Elisabeth TRAIFFORT; Marc DELPECH
- **Personne qualifiée en raison de ses compétences en matière de biostatistique ou d'épidémiologie** : Christophe BARDIN
- **médecin généraliste** : Catherine GRILLOT-COURVALIN
- **Infirmière** : Cécile KORONKIEWICZ ; Jeannine TAILLARD

- DEUXIEME COLLEGE

- **Personne qualifiée en raison de ses compétences juridiques** : Angélique COZETTE
- **Représentant des associations agréées de malades ou d'usagers du système de santé** : Pierre FRANTZ ; Françoise PINSARD ; Marianne BARRIERE
- **Personne qualifiée en raison de sa compétence à l'égard des questions éthiques** : Jean-Michel ZUCKER
- **Travailleur social** : Catherine MAZIN
- **Psychologue** : Magali SEASSAU

COMPOSITION :
Présidente : Dr Elisabeth FRIJA-ORVOEN ; Vice-président : Pr. Jean-Michel ZUCKER ; Secrétaire Scientifique : Christophe BARDIN
Autres membres :
Astrid BARBEY ; Marianne BARRIERE ; Angélique COZETTE ; Pr. Marc DELPECH ; Vianney DESCROIX ; Pierre FRANTZ ; Danielle GOLINELLI ; Dr Catherine GRILLOT-COURVALIN ; Dr Michelle HADCHOUEL ; Cécile KORONKIEWICZ ; Catherine LABRUSSE-RIOU ; Catherine MAZIN ; Dr Jean-Louis PERIGNON ; Françoise PINSARD ; Magali SEASSAU ; Jeannine TAILLARD ; Elisabeth TRAIFFORT ; Dr Jacques TRETON

Désormais, pour toute soumission d'un amendement, le Comité souhaite recevoir ces documents :

- **3 exemplaires papier de l'amendement** sur lesquels sont reportées nos références ainsi que le titre complet de l'étude.
- **Une lettre rédigée en français qui explicite la rationalité de l'amendement**, ainsi que son **impact sur les risques et les contraintes** si l'amendement entraîne une modification du formulaire du consentement et de la notice d'information.
- **une version électronique** de l'ensemble de ces documents sur laquelle sont reportées nos références ainsi que le titre complet de l'étude. (soit par email en ficher joint, soit couchée sur CD ou DVD) sur laquelle sont reportées nos références ainsi que le titre complet de l'étude.
- **S'il s'agit d'un amendement important et qui nécessite beaucoup de modifications dans le corps du texte, joindre la partie du document initial afin que le rapporteur puisse s'y référer et comparer les deux textes.** Pour toute modification ou correction relatives au protocole, à la notice d'information, ou au formulaire de consentement, **bien les mettre en évidence** afin de faciliter aux rapporteurs la relecture des documents (par exemple utiliser une autre couleur, le mode souligné ou italique).

Dr Elisabeth FRIJA
La Présidente du CPP Ile de France 1
