# Supplementary material for: Assessment of a Standardized Pre-Operative Telephone Checklist Designed to Avoid Late Cancellation of Ambulatory Surgery: The AMBUPROG Multicenter Randomized Controlled Trial
Source: PLoS One. 2016 Feb 1;11(2):e0147194. doi: 10.1371/journal.pone.0147194 (PMC4734771; doi:10.1371/journal.pone.0147194)
Supplement: S7 Protocol — (PDF) [file pone.0147194.s008.pdf]

COMITE DE PROTECTION DES PERSONNES - Ile de France 1

CPP ILE DE France I – N°IRB : 00008522 – responsable administrative : Hélène de Crécy
Hôtel-Dieu – 1, Place du Parvis Notre-Dame – 75181 PARIS cedex 04
Tél. : 01 42 34 80 52 – Port. 06 63 34 80 52 – Fax : 01 42 34 86 11 – E-Mail : cppiledefrance1@orange.fr – E-Mail : ccp.prb@htd.aphp.fr

Ludovic DYEN - Chef de Projet
DIRC Ile de France
Assistance Publique-Hôpitaux de Paris
(Direction de la Recherche Clinique et du Développement)
Carré Historique,
Hôpital Saint Louis, Secteur Gris, Porte 23
1 Av. Claude Vellefaux
75475 Paris Cedex 10

Tel: +33 (0)1.44.84.17.43
Fax: +33 (0)1.44 84 17.01
Email: ludovic.dyen@sls.aphp.fr

Paris, le 24 juillet 2012

Nos références CPP Ile de France 1 - NUMERO DOSSIER : 2012- juillet -12991
Amendement n°1 au 2012-Janv.-12806

Le 13 juillet 2012, le comité a été saisi d'une demande initiale concernant le projet de recherche en soins courants intitulé : **AMBUPROG. Impact d'une "check-list" informatisée sur le taux de déprogrammation tardive des patients en chirurgie ambulatoire. Réf. Promoteur : PHRQ1145 – ID RCB 2011-A01647-34**

- **Promoteur** : Assistance Publique – Hôpitaux de Paris
- **Investigateur principal** : Investigateur Principal : Pr Jean-Pierre BETHOUX, Service de Chirurgie Générale Viscérale et Thoracique - Hôpital HOTEL DIEU, 1 PL DU PARVIS NOTRE-DAME, 75004 PARIS

Cette modification substantielle porte sur les points et documents suivants :

- Modification de l'adresse du service du centre Coordinateur : Déménagement su service dans un autre hôpital.
- Modification de la check-list : Modification du circuit de prise en charge de la réponse négative ou positive aux questions pour s'assurer que l'information délivrée par le serveur entraine une action ; en effet le circuit de l'information est hétérogène entre les UCA et les équipes tournent. Un mail envoyé n'avait donc pas l'assurance d'une part d'être lu en temps en en heure d'autre part d'être lu par la personne adéquate.
- Prise en compte de la modification du circuit de prise en charge de la réponse négative ou positive aux questions de la Check-list entrainant l'intervention potentielle du patient, dans les sections 4.2 et 6.3.

Cette demande porte sur les éléments suivants dans les 2 notes d'informations :

➢ Prise en compte de la modification du circuit de prise en charge de la réponse négative ou positive aux questions de la Check-list entrainant l'intervention potentielle du patient.

COMPOSITION :
Président : Christophe BARDIN ; Vice-présidente : Angélique COZETTE ; Secrétaires Scientifiques : Catherine GRILLOT-COURVALIN, Magali SEASSAU ; Trésorière : Elisabeth FRIJA-ORVOEN

Autres membres :
Astrid BARBEY ; Marianne BARRIERE ; Christophe BAZIN ; Nathalie DAFFOS ; Marc DELPECH ; Vianney DESCROIX ; Samuel FITOUSSI ; Pierre FRANTZ ; Danielle GOLINELLI ; Cécile KORONKIEWICZ ; Catherine LABRUSSE-RIOU ; Catherine MAZIN ; Jean-Louis PERIGNON ; Françoise PINSARD ; Marie-France POIRIER ; Jeannine TAILLARD ; Elisabeth TRAIFFORT ; Jacques TRETON ; Jean-Michel ZUCKER

Pièces jointes :

	Documents	Type	Nbre d'ex.	Version et Date
	Courrier de saisine	Original<br>Co[illegible]e	1<br>3	06/07/2012
	Formulaire de demande d'avis au Comité de Protection des Personnes pour une recherche visant à évaluer les soins courants mentionnée au 2° de l'article L, 1121-1 du code de la santé publique, *mise à jour avec suivi de modification*	Copie		V2.0 du 06/07/2012
	Protocole avec *suivi de modifications*	Copie		V 2.0 du 06/07/2012
4.	Note d'information destinée aux adultes, avec *suivi de modification*	Copie	3	V2.0 du 06/07/2012
	Note d'information destinée aux titulaires de l'exercice de l'autorité parentale, avec *suivi de modification*	Copie		V2.0 du 06/07/2012
6.	Tableau de suivi des modifications	Copie		V 1.0 du 06/07/2012

Le Comité adopté ce jour, lundi 23 juillet 2012, la délibération suivante :

AVIS FAVORABLE

Cet avis favorable est valable un an à partir de la date de son émission.

Désormais, pour toute soumission d'un amendement, le Comité souhaite recevoir ces documents :

- 3 exemplaires papier de l'amendement sur lesquels sont reportées nos références ainsi que le titre complet de l'étude.
- **Une lettre rédigée en français qui explicite la rationalité de l'amendement**, ainsi que son **impact sur les risques et les contraintes** si l'amendement entraîne une modification du formulaire du consentement et de la notice d'information.
- **une version électronique** de l'ensemble de ces documents sur laquelle sont reportées nos références ainsi que le titre complet de l'étude. (soit par email en ficher joint, soit couchée sur CD ou DVD) sur laquelle sont reportées nos références ainsi que le titre complet de l'étude.
- **S'il s'agit d'un amendement important et qui nécessite beaucoup de modifications dans le corps du texte, joindre la partie du document initial afin que le rapporteur puisse s'y référer et comparer les deux textes.** Pour toute modification ou correction relatives au protocole, à la notice d'information, ou au formulaire de consentement, **bien les mettre en évidence** afin de faciliter aux rapporteurs la relecture des documents (par exemple utiliser une autre couleur, le mode souligné ou italique).

Christophe BARDIN
Président du CPP Ile de France 1
