# Supplementary material for: Assessment of a Standardized Pre-Operative Telephone Checklist Designed to Avoid Late Cancellation of Ambulatory Surgery: The AMBUPROG Multicenter Randomized Controlled Trial
Source: PLoS One. 2016 Feb 1;11(2):e0147194. doi: 10.1371/journal.pone.0147194 (PMC4734771; doi:10.1371/journal.pone.0147194)
Supplement: S8 Protocol — (PDF) [file pone.0147194.s009.pdf]

COMITE DE PROTECTION DES PERSONNES - Ile de France 1

CPP ILE DE France I – N°IRB : 00008522 – responsable administrative : Hélène de Crécy
Hôtel-Dieu – 1, Place du Parvis Notre-Dame – 75181 PARIS cedex 04
Tél. : 01 42 34 80 52 – Port. 06 63 34 80 52 – Fax : 01 42 34 86 11 – E-Mail : cppiledefrance1@orange.fr – E-Mail : ccp.prb@htd.aphp.fr

Ludovic DYEN - Chef de Projet
DIRC Ile de France
Assistance Publique-Hôpitaux de Paris
(Direction de la Recherche Clinique et du Développement)
Carré Historique,
Hôpital Saint Louis, Secteur Gris, Porte 23
1 Av. Claude Vellefaux
75475 Paris Cedex 10

Tel: +33 (0)1.44.84.17.43
Fax: +33 (0)1.44 84 17.01
Email: ludovic.dyen@sls.aphp.fr

Paris, le 6 septembre 2013

Nos références CPP Ile de France 1 - NUMERO DOSSIER : 2013-sept.-13362
Amendement n°4 au 2012-Janv.-12806

Le 13 août 2013, le comité a été saisi d'une demande complémentaire concernant le projet de recherche en soins courants intitulé : **AMBUPROG. Impact d'une "check-list" informatisée sur le taux de déprogrammation tardive des patients en chirurgie ambulatoire. Réf. Promoteur : PHRQ1145 – ID RCB 2011-A01647-34**

- **Promoteur** : Assistance Publique – Hôpitaux de Paris
- **Investigateur principal** : Investigateur Principal : Pr Jean-Pierre BETHOUX, Service de Chirurgie Générale Viscérale et Thoracique - Hôpital HOTEL DIEU, 1 PL DU PARVIS NOTRE-DAME, 75004 PARIS

Cette modification substantielle porte sur les points et documents suivants :
Changement d'investigateur principal du centre 7/Hôpital Trousseau-Paris : le Dr Auber a quitté le service fin juillet 2013. Il est remplacé par le Dr Delaporte-Cerceau qui était déjà impliqué dans l'étude comme co-investigatrice.

Pièces jointes :

- Courrier de saisine du 31 07 2013
- Formulaire de demande d'avis au comité de protection des personnes pour une recherche visant à évaluer les soins courants mentionnée au 2° de l'article L. 1121-1 du code de la santé publique, mise à jour avec suivi de modification, version 1.0 du 31 07 2013
- Addendum 1.0 au protocole 4.0 du 20 06 2013 avec suivi des modifications
- Cv du Dr Delaporte-Cerceau

Le BUREAU a adopté ce jour, mercredi 4 septembre 2013, la délibération suivante :

AVIS FAVORABLE

Ont participé à la délibération :

- PREMIER COLLEGE

- **Médecin ou personne qualifiée en matière de recherche biomédicale** : Elisabeth TRAIFFORT; Danielle GOLINELLI ; Elisabeth FRIJA ; Marc DELPECH ; Vianney DESCROIX
- **Personne qualifiée en raison de ses compétences en matière de biostatistique ou d'épidémiologie** : Christophe BARDIN
- **Médecin généraliste** : Jean-Louis PERIGNON ; Catherine GRILLOT-COURVALIN
- **Infirmière** : Cécile KORONKIEWICZ ; Jeannine TAILLARD
- **Pharmacien hospitalier** : Annick TIBI

COMPOSITION :
Président : Christophe BARDIN ; Vice-présidente : Angélique COZETTE ; Secrétaires Scientifiques : Catherine GRILLOT-COURVALIN, Magali SEASSAU ; Trésorière : Elisabeth FRIJA-ORVOEN

Autres membres :
Astrid BARBEY ; Marianne BARRIERE ; Christophe BAZIN ; Nathalie DAFFOS ; Marc DELPECH ; Vianney DESCROIX ; Samuel FITOUSSI ; Pierre FRANTZ ; Danielle GOLINELLI ; Cécile KORONKIEWICZ ; Catherine LABRUSSE-RIOU ; Catherine MAZIN ; Jean-Louis PERIGNON ; Françoise PINSARD ; Marie-France POIRIER ; Jeannine TAILLARD ; Annick TIBI ; Elisabeth TRAIFFORT ; Jacques TRETON ; Jean-Michel ZUCKER

- <u>DEUXIEME COLLEGE</u>

- **Personne qualifiée en raison de ses compétences juridiques** : Catherine LABRUSSE-RIOU
- **Psychologue** : Magali SEASSAU
- **Représentant des associations agréées de malades ou d'usagers du système de santé** : Marianne BARRIERE ; Pierre FRANTZ ; Françoise PINSARD
- **Travailleur social** : Catherine MAZIN

Désormais, pour toute soumission d'un amendement, le Comité souhaite recevoir ces documents :

- **<u>3 exemplaires papier de l'amendement</u>** sur lesquels sont reportées nos références ainsi que le titre complet de l'étude.
- **<u>Une lettre rédigée en français qui explicite la rationalité de l'amendement</u>**, ainsi que son **<u>impact sur les risques et les contraintes</u>** si l'amendement entraîne une modification du formulaire du consentement et de la notice d'information.
- **<u>une version électronique</u>** de l'ensemble de ces documents sur laquelle sont reportées nos références ainsi que le titre complet de l'étude. (soit par email en ficher joint, soit couchée sur CD ou DVD) sur laquelle sont reportées nos références ainsi que le titre complet de l'étude.
- **S'il s'agit d'un amendement important et qui nécessite beaucoup de modifications dans le <u>corps du texte, joindre la partie du document initial</u> afin que le rapporteur puisse s'y référer et comparer les deux textes.** Pour toute modification ou correction relatives au protocole, à la notice d'information, ou au formulaire de consentement, **<u>bien les mettre en évidence</u>** afin de faciliter aux rapporteurs la relecture des documents (par exemple utiliser une autre couleur, le mode souligné ou italique).

Dr Christophe BARDIN
Président du CPP Ile de France 1

P (·
